# Enlarged PML-nuclear bodies trigger conflicting cell cycle signal-mediated cytotoxicity in leukemia cells

**DOI:** 10.1038/s41419-025-07911-7

**Published:** 2025-08-02

**Authors:** Tomohisa Baba, Soichiro Kumamoto, Yuta Moriguchi, Soji Morishita, Atsushi Hirao, Yoshikazu Johmura

**Affiliations:** 1 Division of Cancer and Senescence Biology, Cancer Research Institute, Kanazawa University, Kanazawa, Ishikawa Japan; 2 Department of Advanced Hematology, Juntendo University Graduate School of Medicine, Tokyo, Japan; 3 Division of Molecular Genetics, Cancer Research Institute, Kanazawa University, Kanazawa, Ishikawa Japan; 4 Integrated Systems of Aging Research Unit, Institute for Frontier Science Initiative, Kanazawa University, Kanazawa, Ishikawa Japan

**Keywords:** PML bodies, Apoptosis

## Abstract

Accumulating evidence suggests that mitogenic signaling during cell cycle arrest can lead to severe cytotoxic outcomes, such as senescence, though the underlying mechanisms remain poorly understood. Here, we explored the link between cell cycle dynamics and the formation of PML-nuclear bodies (PML-NBs), intranuclear structures known to mediate cellular stress responses. Our findings demonstrate that PML-NBs increase their number during interphase arrest. Moreover, the activation of mitogenic ERK signaling by all-trans retinoic acid (ATRA) during CDK4/6 inhibitor-induced cell cycle arrest synergistically enhances the formation of larger PML-NBs by associating with SUMO. This enlargement, triggered by the simultaneous engagement of opposing cell cycle signals, leads to potent cytotoxicity accompanied by either terminal differentiation or apoptosis, depending on the cell type, across multiple acute myeloid leukemia (AML) cell lines. Importantly, in an AML mouse model, this combination treatment significantly improved therapeutic efficacy with minimal effects on normal hematopoiesis. Our results introduce conflicting cell cycle signal-induced cytotoxicity as a promising therapeutic strategy for AML.

## Introduction

Cellular fate decisions, such as differentiation, apoptosis, and senescence, are closely linked to cell cycle exit. However, whether the cell cycle inversely regulates these processes remains unclear. In cellular senescence, paradoxical activation of growth signaling in non-proliferating cells leads to disruption of the cell cycle, triggering senescence [1]; however, the underlying molecular mechanisms are not yet fully understood. PML-nuclear bodies (PML-NBs), which serve as scaffolds for epigenetic regulation [2], play critical roles in cellular stress responses, particularly in the induction of apoptosis and senescence [3–5]. Moreover, PML-NB formation is tightly regulated by the cell cycle [6], suggesting a reciprocal interaction between cell cycle progression and cellular fate decisions via PML-NBs.

Acute myeloid leukemia (AML) is driven by various oncogenic mutations that complicate the development of therapeutic strategies broadly applicable across different AML subtypes. Nevertheless, accumulating evidence suggests that many leukemogenic agents directly or indirectly inhibit normal myeloid differentiation of leukemia cells at immature stages [7]. Notably, the PML-RARA fusion protein promotes the expansion of leukemia cells by obstructing normal myeloid differentiation via the retinoic acid receptor (RAR) signaling pathway in acute promyelocytic leukemia (APL) [8, 9]. Conversely, all-trans retinoic acid (ATRA), which activates RAR signaling, and arsenic trioxide (ATO) effectively induce myeloid differentiation and inhibit the proliferation of PML-RARA-expressing APL cells [10, 11]. Consequently, the therapeutic potential of pharmacological agents capable of promoting myeloid differentiation in leukemia cells were extensively explored, leading to the development of a treatment strategy known as differentiation therapies [7]. Among these agents, ATRA and ATO represent the most significant advancements in clinical hematology.

ATRA functions as a transcriptional activator of RAR-regulated genes and promotes the degradation of PML-RARA [12]. The former primarily enhances the differentiation of APL cells, while the latter reduces their leukemia-initiating capacity [12]. As the PML-RARA fusion protein is degraded, the normal PML protein, which was previously sequestered within the PML-RARA complex, is released. This release allows the normal PML protein to reassemble into PML-NBs in the nucleoplasm, which is essential for its therapeutic effect in APL [13]. Conversely, while many studies have shown that ATRA promotes myeloid differentiation in various types of AML cells, few have demonstrated its clinical benefits in patients with non-APL leukemia [14–17]. Therefore, activation of PML-NB formation is considered a crucial step in achieving the full therapeutic effect of differentiation therapy.

In this study, we screened for more effective and broadly applicable therapeutic strategies against AML, using PML-NB formation as a hallmark. As expected, treatment with ATRA alone significantly increased PML-NBs in NB4 cells, a human APL cell line (M3 in the FAB classification), but not in the PML-RARA-negative HL60 cell line, which shares similar differentiation characteristics (M2) [18]. Notably, the combination of ATRA with the CDK4/6 inhibitor (CDK4/6i) palbociclib induces enlarged PML-NB-dependent cytotoxicity through conflicting cell cycle signals, driven by ATRA-dependent activation of mitogenic ERK signaling under conditions of cell cycle arrest in non-APL leukemia. Given the clear synergistic therapeutic effects observed in AML-bearing mice treated with this combination, we propose a therapeutic strategy for AML based on this novel cell biological phenomenon.

## Results

### Single ATRA treatment is effective in NB4 APL cell line with a concurrent increase in intranuclear PML-NBs

**Fig. 1:**
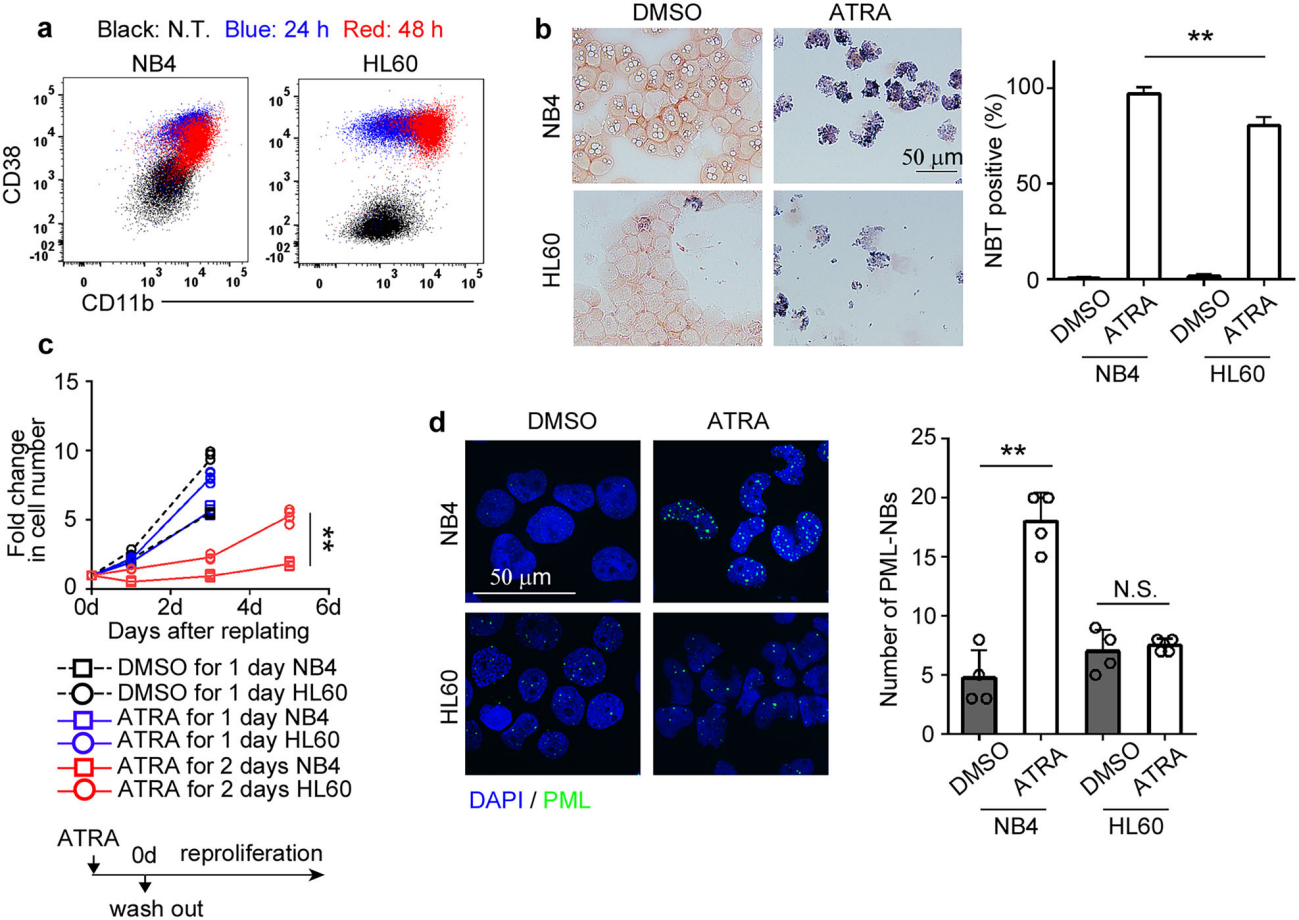
ATRA-induced myeloid differentiation and PML-NB formation in APL cell line NB4. NB4 and HL60 cell lines were treated with 1 μM ATRA. **a** The expression of CD38 and CD11b on non-treated cells (black dots) and cells treated with ATRA for 24 h (blue dots) or 48 h (red dots) are shown. Representative data from three independent experiments are shown. **b** NBT staining of NB4 and HL60 cells 48 h after treatment with ATRA. DMSO-treated cells served as controls. Representative images and mean + SD of the percentage of NBT-positive cells, calculated from eight randomly selected fields across three independent experiments, are shown. **c** Cell growth in drug-free medium following prior incubation with ATRA for 24 or 48 h (*n* = 4). DMSO-treated cells for 24 h served as controls. The fold change in cell number was calculated by dividing the values at each time point by the values at day 0. **d** Immunofluorescent staining for PML-NBs in NB4 and HL60 cells 48 h after treatment with ATRA. DMSO-treated cells were used as controls. Representative images and mean + SD of the number of PML-NBs per nucleus are shown (*n* = 4). **P < 0.01; N.S., no significant difference (two-sided Student’s *t*-test).

Consistent with previous reports [19], ATRA treatment alone induced myeloid differentiation, as evidenced by the expression of myeloid cell markers CD38 and CD11b, and increased nitro blue tetrazolium (NBT) staining. This effect was more pronounced in PML-RARA^+^ NB4 cells compared with that in PML-RARA^-^ HL60 cells (Fig. 1a, b). Notably, the late differentiation phenotype [20, 21] characterized by CD11b^high^CD38^low^ was exclusively induced in NB4 cells (Fig. 1a). Furthermore, a 48 h-pre-exposure to ATRA, but not a 24 h-pre-exposure, efficiently inhibited cell proliferation even after the drug was removed, with a greater effect observed in NB4 cells (Fig. 1c). This enhanced therapeutic effect against APL cells correlated with the observation that ATRA treatment specifically enhanced the number and signal intensity of PML-NBs in NB4 cells (Fig. 1d and Supplementary Fig. 1).

### PML-NBs increased with cell cycle arrest during interphase

**Fig. 2:**
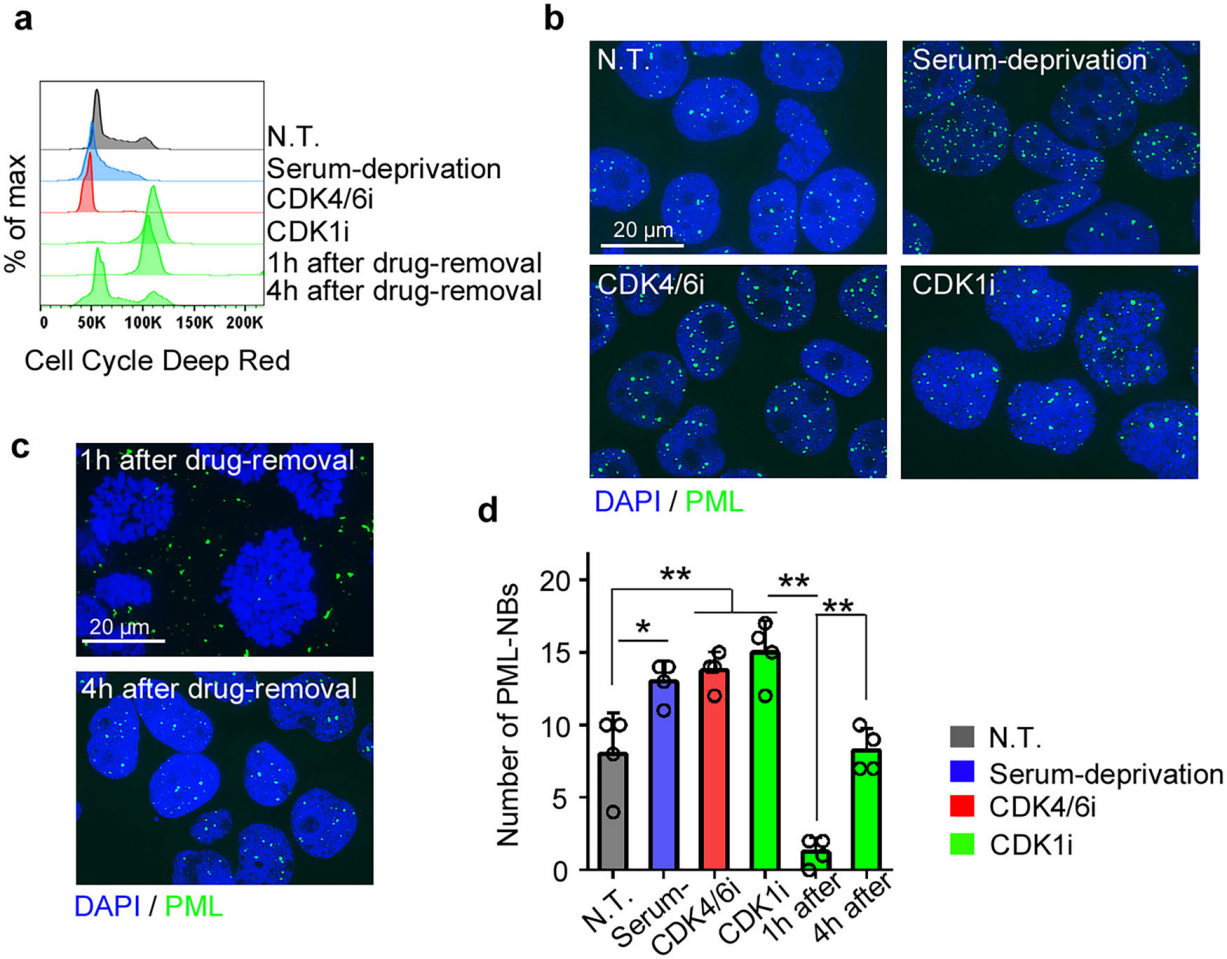
PML-NBs increased with cell cycle arrest during interphase in HL60 cells. **a** Cell cycle status was assessed 24 h post-treatment with CDK4/6i, CDK1i, or serum-free medium, as well as at the indicated time points following incubation in drug-free medium after a 24 h prior treatment with CDK1i. Representative data from three independent experiments are shown. **b**, **c** Immunofluorescent staining for PML-NBs was performed at the time points indicated in (**a**). Representative images and the mean + SD of the number of PML-NBs per nucleus are shown in **d** (*n* = 4). **P < 0.01; *P < 0.05 (Tukey–Kramer’s test).

The formation of PML-NBs is closely coordinated with cell cycle progression. While PML-NBs are primarily localized within the nucleus during interphase, they are expelled into the cytoplasm due to nuclear membrane breakdown upon entry into mitosis. In the post-mitotic stage, their nuclear import and reconstitution can be observed [6]. To investigate the relationship between PML-NBs and cell cycle arrest, we examined PML-NB formation under various cell cycle inhibition conditions (Fig. 2a). Serum deprivation (G0/G1 arrest), treatment with the CDK4/6 inhibitor palbociclib (G1 arrest), and the CDK1 inhibitor RO3306 (G2 arrest), resulted in a comparable increase in PML-NB numbers (Fig. 2b, d). Consistent with previous studies [6], most PML-NBs were expelled from the nucleus in mitotic cells 1 h after CDK1 inhibition was removed, with subsequent spontaneous reassembly within the nucleoplasm upon transition to the G1 phase (Fig. 2a, c, d). These findings collectively suggest that cell cycle arrest during interphase promotes the continuous reassembly of PML-NBs.

### Synergistic damage to non-APL cells by CDK4/6 inhibitor palbociclib and ATRA through a distinctive PML-NB formation pattern

**Fig. 3:**
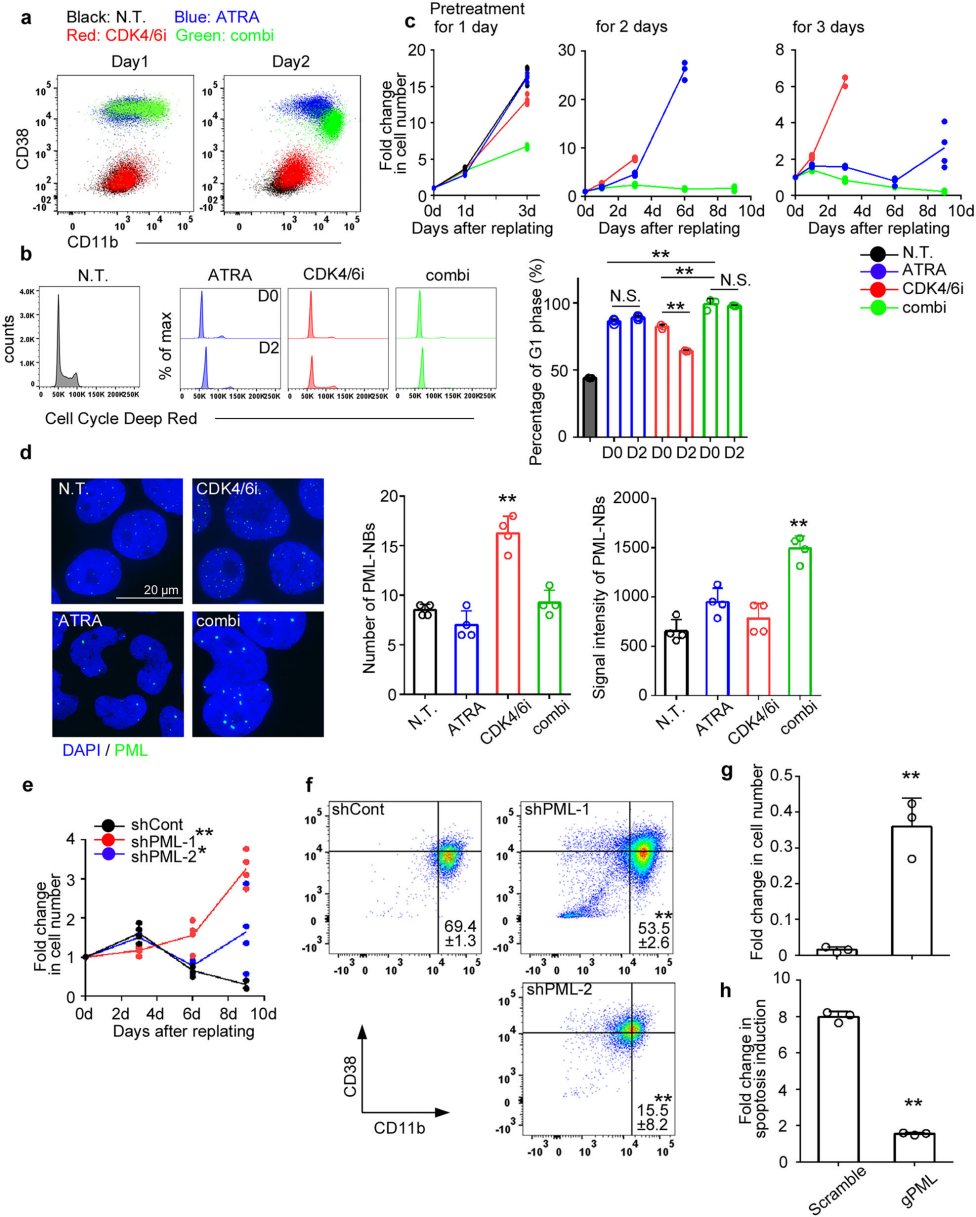
Combined treatment with CDK4/6i and ATRA induces the irreversible cell growth arrest in HL60 cells through a distinctive pattern of PML-NB formation. HL60 cells were treated with 1 μM CDK4/6i and/or 1 μM ATRA, with non-treated cells serving as controls. **a** The expression levels of CD38 and CD11b in HL60 cells were assessed at 24 h and 48 h post-treatment with CDK4/6i (red dots), ATRA (blue dots), or the combination of both drugs (green dots). Non-treated cells are represented by black dots. Representative data from three independent experiments are shown. **b** Cell cycle status was evaluated after drug treatment and 48 h after drug removal. Representative images and mean + SD of percentage of cells in G1 phase are shown (*n* = 3). **c** Cell growth was measured in drug-free medium following the indicated period of prior drug treatment (*n* = 4). The fold change in cell number was calculated by dividing the values at each time point by the values at day 0. **d** Immunofluorescent staining for PML-NBs was performed 48 h after drug treatment. Representative images and the mean + SD of the number of PML-NBs per nucleus and the average of their signal intensity are shown (*n* = 4). **e** Proliferation of shControl- and shPML-transduced HL60 cells was assessed in drug-free medium following 48 h of prior CDK4/6i+ATRA treatment (*n* = 4). The fold change in cell number was calculated by dividing the values at each time point by the values at day 0. **f** The expression of CD38 and CD11b on shControl- and shPML-transduced HL60 cells 48 h after CDK4/6i+ATRA treatment. The mean ± SD of the percentage of CD11b^high^CD38^low^ cells is shown (*n* = 3). **g**, **h** Cell number and apoptosis induction were assessed in PML-specific gRNA transduced MOLM-13 cells 48 h after CDK4/6i+ATRA treatment. The fold changes were calculated by dividing with the values of non-treated cells. The mean + SD from three independent experiments is shown. **P < 0.01; *P < 0.05; N.S., no significant difference (Tukey–Kramer’s test for (**b**); Dunnett’s test for (**d**, **e**); two-sided Student’s *t*-test for (**g**, **h**).

Although CDK4/6i has been clinically approved for breast cancer treatment [22], CDK4/6i monotherapy did not induce cytotoxicity in HL60 cells, as evidenced by the maintenance of their immature state (Fig. 3a) and their rapid re-entry into the cell cycle after the drug was removed (Fig. 3b). We previously demonstrated that autophagy inhibitors promote ATRA-mediated myeloid differentiation in non-APL cells by inducing the accumulation of the CDK inhibitor p21 protein through inhibition of its proteasomal degradation [21]. In support of this, a recent study showed that palbociclib synergizes with ATRA to enhance myeloid differentiation of HL60 cells [23]. Consistent with these findings, we observed that CDK4/6i obviously enhanced myeloid differentiation when combined with ATRA, as indicated by the downregulation of CD38 on CD11b^high^ cells, which became apparent two days after the combination treatment (Fig. 3a). Furthermore, the drug treatments induced G1-phase cell cycle arrest, with a more pronounced effect observed for the combined treatment with CDK4/6i and ATRA (CDK4/6i+ATRA); this arrest was not reversible 48 h after the drug was removed (Fig. 3b). Notably, 48- and 72 h-pre-exposure to CDK4/6i+ATRA irreversibly inhibited cell proliferation, even after the drug was washed out (hereafter referred to as irreversible cell growth arrest), whereas pre-exposure to either ATRA or CDK4/6i failed to do so (Fig. 3c). This irreversible cell cycle arrest was further supported by the activation of β-galactosidase, a marker of cellular senescence, which was preferentially induced by the combination treatment in HL60 cells (Supplementary Fig. 2a). Incidentally, neither CDK4/6i monotherapy nor its combination with ATRA (CDK4/6i+ATRA) directly induced cell death in HL60 cells, despite their ability to suppress proliferation (Supplementary Fig. 2b, c). RNA-Seq analysis revealed that CDK4/6i+ATRA exerted synergistic, rather than additive effects on HL60 cells (Supplementary Fig. 2d, e), characterized by the upregulation of immune activation– and senescence-related genes, and downregulation of cell cycle–related genes (Supplementary Fig. 2f, g). Although CDK4/6i+ATRA did not significantly enhance myeloid differentiation or induce irreversible cell growth arrest (Supplementary Fig. 3), it preferentially triggered apoptotic cell death in the human acute monoblastic and monocytic leukemia cell lines, MV4-11, MOLM-13, and MOLM-14 (classified as M5 in the FAB classification [18]) (Supplementary Figs. 4 and 5a–c).

In addition to these potent anti-leukemic effects, we observed a distinctive pattern of PML-NB formation in HL60 cells treated with the drug combination. Notably, the increase in the number of PML-NBs was independent of the enhancement in signal intensity; while the number of PML-NBs increased with CDK4/6i treatment alone, the signal intensity was augmented specifically with CDK4/6i+ATRA (Fig. 3d). To investigate the contribution of PML-NBs to the anti-leukemic effects, PML expression was silenced using shRNAs in HL60 cells (Supplementary Fig. 6a). shPML treatment significantly attenuated CDK4/6i+ATRA-mediated irreversible cell growth arrest (Fig. 3e) and myeloid differentiation (Fig. 3f). Furthermore, CDK4/6i+ATRA-induced apoptosis in MOLM-13 cells, which concomitantly exhibited enhanced PML-NB signal intensity (Supplementary Fig. 5d), was inhibited by PML-specific gRNA transduction using the CRISPR-Cas9 system (Fig. 3g, h, and Supplementary Fig. 6b).

### Increased PML-NBs under cell cycle arrest are multimerized by mitogenic ERK signaling

**Fig. 4:**
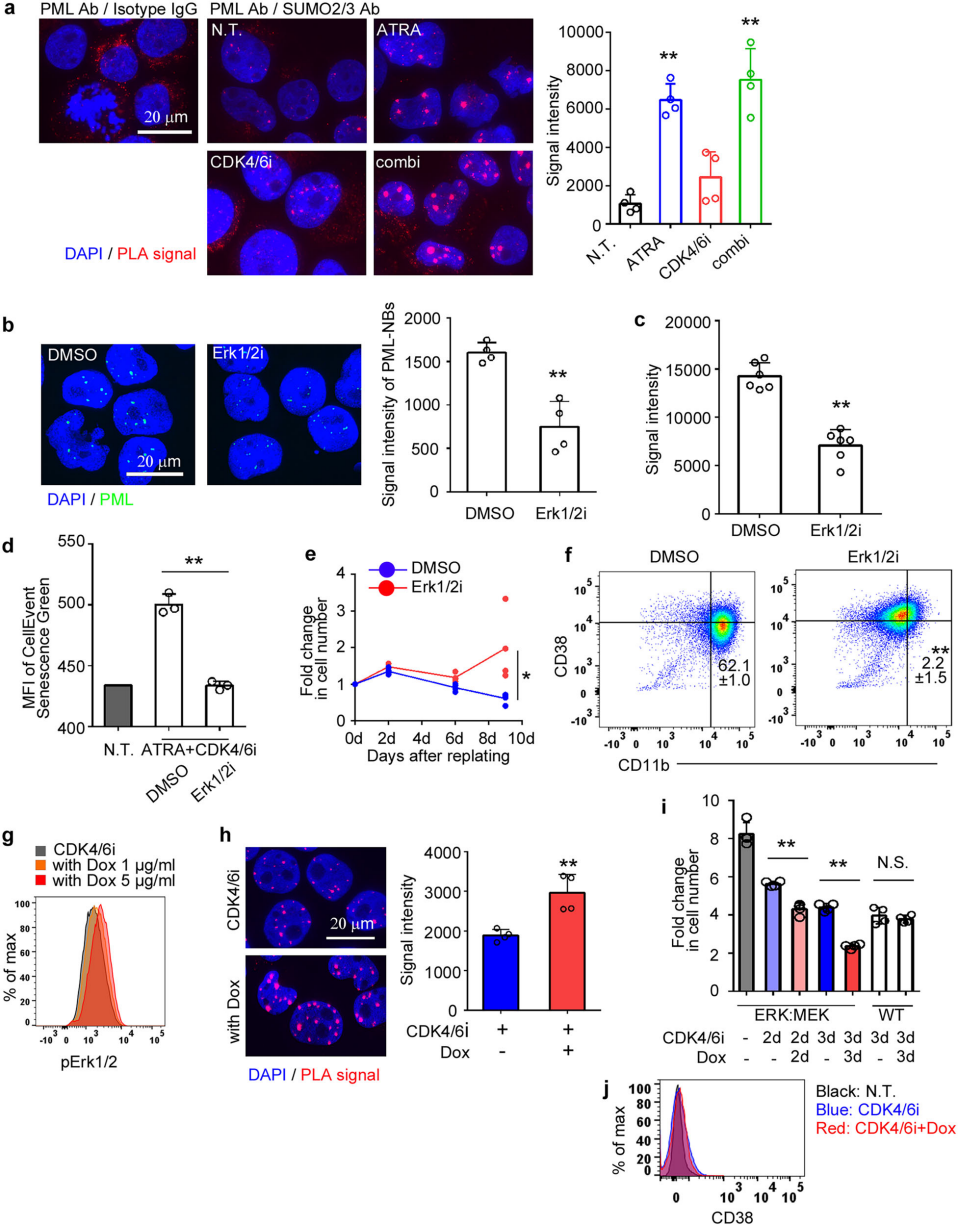
Increased PML-NBs under conditions of cell cycle arrest are multimerized by mitogenic ERK signaling. **a** Direct interaction of PML with SUMO was assessed at 48 h post-treatment with ATRA, CDK4/6i, or the combination of both drugs using PLA in HL60 cells. Representative images and the mean + SD of the signal intensity of PLA foci are shown (*n* = 4). HL60 cells were pre-treated with 1 μM ERK1/2i for 10 min, followed by treatment with CDK4/6i+ATRA in the presence (**b**, **c**, **d**, and **f**) or absence (**e**) of 1 μM ERK1/2i. Immunofluorescent staining was performed to detect PML-NBs (**b**), Direct interaction of PML with SUMO was assessed (**c**), β-galactosidase activity was measured (**d**), cell growth was evaluated after drug removal (**e**), and the expression of CD38 and CD11b was analyzed 48 h after drug treatment (**f**). Representative images and/or the means + SD are shown, which were calculated from three independent experiments (**d**, **f**), four independent experiments (**b**, **e**), and six randomly selected fields across three independent experiments (**c**). **g** Phosphorylation of ERK1/2 was examined 24 h after doxycycline treatment in HL60 cells transduced with pCWXPGR-pTF-ERK:MEK. Transfected HL60 cells were treated with CDK4/6i in the presence or absence of 5 μg/ml doxycycline. **h** Direct interaction of PML with SUMO was assessed 24 h after drug treatment, with representative images and the mean + SD of the signal intensity of PLA foci presented. **i** Cell proliferation was measured 48 h after drug removal, and the fold change in cell number was calculated relative to day 0. Parental HL60 cells were used as a wild type (WT) control. The means + SD were calculated from four independent experiments. **j** CD38 expression was assessed 72 h after drug treatment. **P < 0.01; *P < 0.05; N.S. no significant difference (Dunnett’s test for **a**; two-sided Student’s *t*-test for (**b–f**, and **h**; Tukey–Kramer’s test for **i**).

The multivalent binding of a small ubiquitin-like modifier (SUMO) and SUMO-interacting motifs (SIMs) facilitates liquid-liquid phase separation (LLPS), driving the formation of molecular condensates, including PML-NBs [24]. Consistent with a previous report [25], the large PML-NBs observed after treatment with CDK4/6i+ATRA were dissolved in 1,6-hexanediol (Supplementary Fig. 7a–c), a compound known to disrupt LLPS-mediated intracellular condensates [26]. Consistent with the immunocytochemical staining results, western blotting showed that both CDK4/6i and CDK4/6i+ATRA treatments increased the RIPA-insoluble form of the PML protein (Supplementary Fig. 7d), which predominantly consisted of multimerized PML-NBs [25]. Notably, higher molecular weight proteins were observed in cells treated with CDK4/6i+ATRA (Supplementary Fig. 7e). The SUMO inhibitor ML792 reduced the number of PML-NBs while reciprocally increasing their signal intensity (Supplementary Fig. 8a) consistently with the previous report [25]. In the presence of ML792, ATRA failed to further enhance PML-NB signals when combined with CDK4/6i (Supplementary Fig. 8b), suggesting that SUMOylation modulation contributes to this PML-NB remodeling. Finally, we confirmed that ATRA significantly enhanced the molecular interaction of PML with SUMO using the proximity ligation assay (PLA) (Fig. 4a).

The proliferation of HL60 cells was efficiently inhibited by the ERK1/2 inhibitor, ravoxertinib (ERK1/2i), compared to the p38 and mTOR inhibitors (Supplementary Fig. 9a). Consistent with this finding, phosphorylated ERK1/2 was observed under normal culture conditions and further enhanced by ATRA treatment (Supplementary Fig. 9b). ERK1/2-mediated phosphorylation of PML promotes PML SUMOylation [27]. Consistent with these observations, the enhanced signal intensity of PML-NBs and PML-SUMO PLA induced by CDK4/6i+ATRA were significantly attenuated upon treatment with ERK1/2i (Fig. 4b, c). Furthermore, ERK1/2i inhibited the CDK4/6i+ATRA-induced increase in β-galactosidase activity (Fig. 4d), irreversible cell growth arrest (Fig. 4e), and myeloid differentiation (Fig. 4f). To directly confirm the contribution of ERK signaling, we generated HL60 cells transduced with pCWXPGR-pTF-ERK:MEK, which encodes a fusion protein of rat ERK2 and human MEK1 that spontaneously activates ERK signaling [28]. Doxycycline-induced phosphorylation of ERK was observed in a dose-dependent manner (Fig. 4g). Notably, treatment with doxycycline in combination with the CDK4/6i substantially enhanced PML-SUMO PLA signals (Fig. 4h) and inhibited post-treatment cell proliferation (Fig. 4i), but did not promote differentiation (Fig. 4j). Thus, the enlarged PML-NBs can be a hallmark of conflicting cell cycle signals, driven by the activation of mitogenic ERK signaling under conditions of cell cycle arrest, contributing to the observed anti-AML effects.

### Recruitment of histone chaperones into PML-NBs leads to detrimental reduction in de novo protein synthesis

**Fig. 5:**
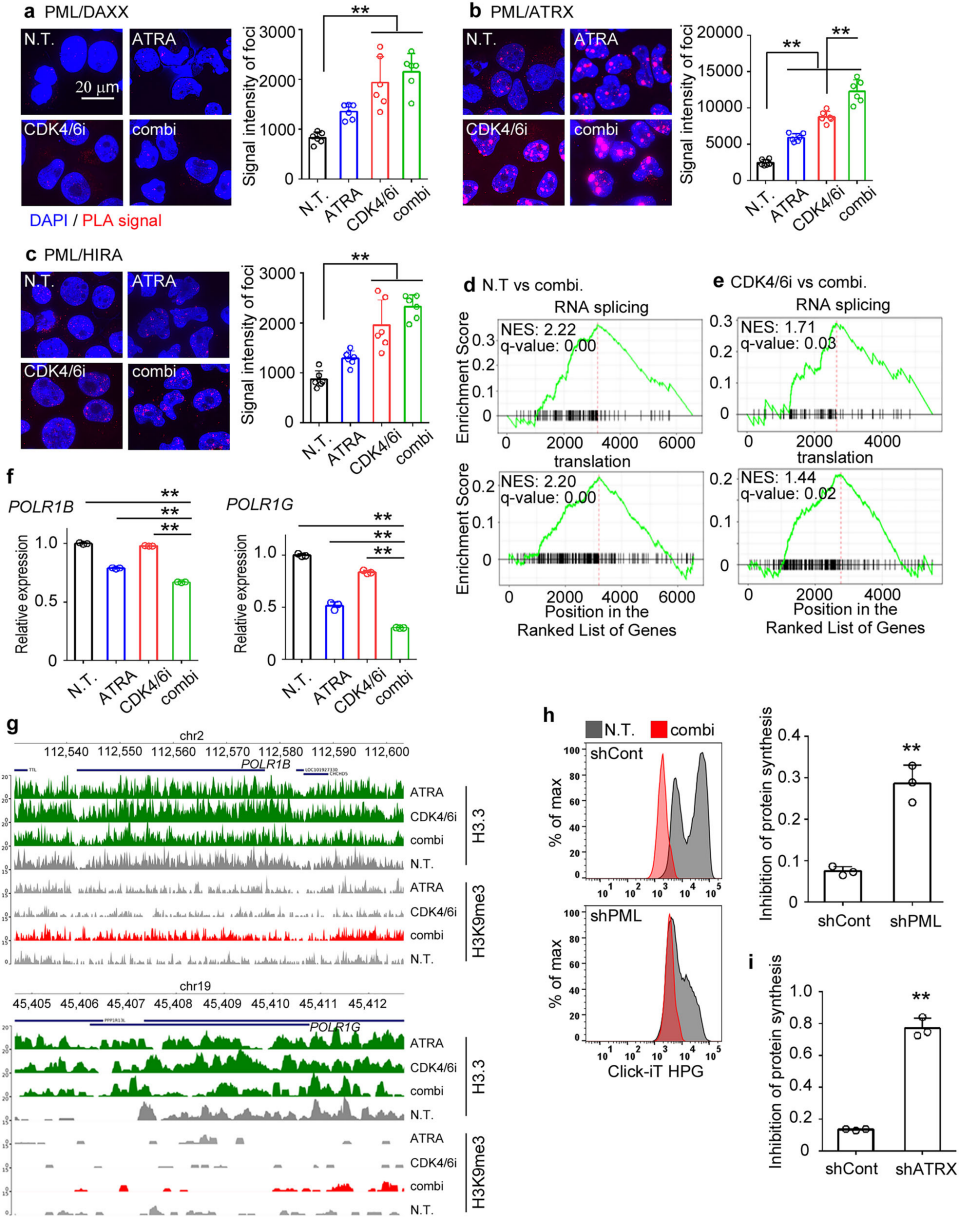
Combined treatment with CDK4/6i and ATRA suppresses de novo protein synthesis and concurrent recruitment of histone chaperones into PML-NBs. HL60 cells were treated with CDK4/6i and/or ATRA, with non-treated cells serving as controls. Molecular interactions of DAXX (**a**), ATRX (**b**), and HIRA (**c**) with PML were assessed 48 h after drug treatment using PLA. Representative images and the means + SD from six randomly selected fields across three independent experiments are shown. **d**, **e** Gene Set Enrichment Analysis (GSEA) was performed using gene sets associated with de novo protein synthesis-related GO terms (“RNA splicing” and “translation”). Comparisons were made between non-treated cells and combined drug-treated cells (**d**), as well as between CDK4/6i-treated cells and combined drug-treated cells (**e**). Normalized enrichment scores (NES) and *q*-values were calculated from three independent experiments. **f** Relative mRNA expression levels of POLR1B and POLR1G (*n* = 3). **g** Chromatin deposition of H3.3 and H3K9me3 at the POLR1B and POLR1G loci. Data representing the deposition at the transcription start site (TSS) regions, which were absent in control cells, are highlighted in green (H3.3) and red (H3K9me3). **h**, **i** De novo protein synthesis in shControl-, shPML-, and shATRX-transduced cells was assessed 48 h after CDK4/6i+ATRA treatment using the Click-iT HPG Alexa Fluor Protein Synthesis Assay. Inhibition of protein synthesis was calculated by dividing the MFI of drug-treated cells by that of untreated cells. Representative data and the mean + SD are shown (*n* = 3). **P < 0.01 (Tukey–Kramer’s test for **a**–**c** and **f**; two-sided Student’s *t*-test for **h**, **i**).

In NB4 cells, ATRA treatment has recently been reported to induce cellular senescence through the recruitment of histone chaperones into PML-NBs and the senescence-associated deposition of the histone variant H3.3 [29]. We investigated the recruitment of the DAXX/ATRX complex and HIRA into PML-NBs, both of which are capable of driving histone H3.3 deposition [30]. CDK4/6i+ATRA synergistically enhanced the colocalization of histone chaperons with PML, with more pronounced recruitment observed for ATRX (Fig. 5a–c). Similar to the effects observed with shPML treatment, shATRX treatment significantly attenuated CDK4/6i+ATRA-mediated irreversible cell growth arrest and myeloid differentiation (Supplementary Fig. 10).

DAXX/ATRX is known to epigenetically regulate transcription by promoting histone H3.3 replacement on chromatin throughout the cell cycle [30]. To comprehensively investigate the chromatin deposition of histone H3.3, we performed ChIP-Seq analysis. As shown in Supplementary Fig. 11a, H3.3 ChIP-Seq peaks were broadly distributed across the genome, including genic and intergenic regions. Under different drug treatment conditions, we observed variable changes in H3.3 deposition patterns (Supplementary Fig. 11b). Notably, GO enrichment analysis revealed that more substantial functional terms were identified when using ChIP-Seq peaks commonly observed in both CDK4/6i- and CDK4/6i+ATRA-treated samples (Supplementary Fig. 11c). Interestingly, many of these terms were related to cellular processes such as the cell cycle and transcription/translation. Furthermore, terms associated with transcription/translation were further highlighted when analyzing ChIP-Seq peaks specifically observed in CDK4/6i+ATRA-treated cells (Supplementary Fig. 11c). Transcriptomic analysis consistently revealed that the expression of mRNAs associated with GO terms such as RNA splicing and translation was decreased in CDK4/6i+ATRA-treated HL60 cells compared to non-treated cells (Fig. 5d) or cells treated with CDK4/6i alone (Fig. 5e). Furthermore, CDK4/6i+ATRA cooperatively suppressed the expression of mRNAs encoding RNA polymerase I subunits, POLR1B and POLR1G, which are essential for ribosomal biogenesis (Fig. 5f). Notably, the deposition of histone H3.3 was similarly induced by either drug alone or in combination, whereas H3K9me3 enrichment at transcription start sites of these genes was specifically observed following the combination treatment (Fig. 5g). This suggests that there are potential alterations in the epigenetic regulation of these genes. Consistent with these observations, CDK4/6i+ATRA treatment markedly suppressed de novo protein synthesis in HL60 cells; however, this suppression was significantly attenuated by shPML (Fig. 5h) or shATRX treatment (Fig. 5i). HL60 cells carry a mutation in the *p53* gene [31] that is not commonly observed in AML. In contrast, all M5-classified AML cell lines in which apoptotic cell death was induced following CDK4/6i+ATRA treatment (Supplementary Fig. 5) possessed the wild-type *p53* gene [32]. In MOLM-13 cells, X-ray treatment rapidly increased the total amount of p53 and its various activating modifications. However, neither effect was induced by CDK4/6i+ATRA treatment (Supplementary Fig. 12a). Similar to the observation in HL60 cells, CDK4/6i+ATRA suppressed protein synthesis through a PML-dependent mechanism in MOLM-13 cells (Supplementary Fig. 12b), suggesting that this p53-independent effect is a common cytotoxic event in AML cells.

### Anti-AML cell specific therapeutic effect of combined treatment with CDK4/6i and ATRA in the AML mouse model

**Fig. 6:**
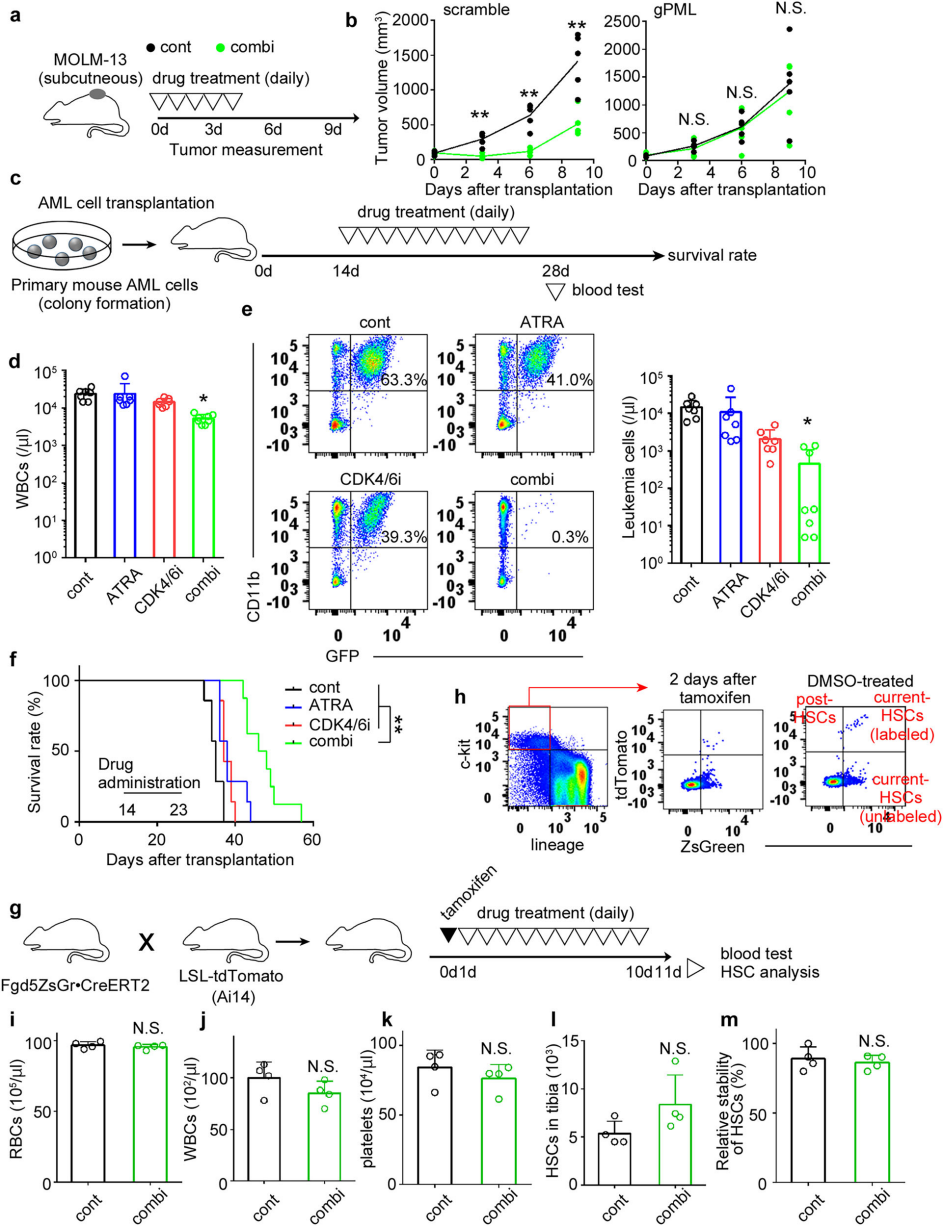
Anti-AML cell specific therapeutic effect of combined treatment with CDK4/6i and ATRA in the AML mouse model. **a** Schematic representation of the experimental design for the combined treatment with CDK4/6i and ATRA in nude mice bearing subcutaneous tumor of scramble- or gPML-transduced MOLM-13. Each treatment group consisted of five mice. **b** The size was calculated using the formula (volume = length × width^2^ /2) and is presented accordingly. **c** Schematic representation of the experimental design for the combined treatment with CDK4/6i and ATRA in mice bearing AML. Seven mice were treated with DMSO (control), CDK4/6i alone, or ATRA alone. Eight mice received the combination treatment. **d** The number of WBCs and (**e**) GFP^+^ AML cells in the PB were evaluated 28 days after AML cell transplantation. **f** Survival rates of mice bearing AML. **g** Schematic representation of the experimental design for the combined treatment with CDK4/6i and ATRA in healthy mice. Four mice were treated with either DMSO or the combination of CDK4/6i and ATRA. **h** Gating strategy for classifying each indicated HSC population, adjusted based on representative data obtained 2 days after tamoxifen injection. The number of RBCs (**i**), WBCs (**j**), and platelets (**k**) were evaluated 24 h after the last drug administration. **l** The number of HSCs was calculated as follows: total BM cells × proportion of lineage^-^c-kit^+^ZsGeen^+^ cells. **m** HSC stability was calculated using the following formula: the number of tdTomato^+^ZsGreen^+^ cells / (the number of tdTomato^+^ZsGreen^+^ cells + tdTomato^+^ZsGreen^−^ cells). **P < 0.01; *P < 0.05; N.S. no significant difference (Dunnett’s test for **d** and **e**; log-rank test for **f**; two-sided Student’s *t*-test for **b**, **i**–**m**).

The in vitro anti-AML effects prompted us to conduct a preclinical study to evaluate the therapeutic efficacy of combined CDK4/6i and ATRA treatment in vivo. To assess whether the cytotoxic effects of the combined drug treatment are dependent on PML in vivo, we established tumor-bearing nude mice using either control or *PML* gene-edited MOLM-13 cells and administered CDK4/6i and ATRA (Fig. 6a). The combination treatment selectively suppressed the tumor growth of PML-intact cells (Fig. 6b). To evaluate the anti-leukemia effects of CDK4/6i and/or ATRA, drug administration began 14 days post-transplantation of primary AML cells derived from mice, coinciding with the emergence of GFP^+^ (MLL-AF9^+^) cells in the peripheral blood (PB) of the recipients, as outlined in the schedule (Fig. 6c). A significant reduction in WBCs and GFP^+^ leukemia cells in the PB was exclusively observed in mice receiving the combination treatment, with no comparable effects observed with monotherapy (Fig. 6d, e). Leukemia cells were almost eradicated from the PB in more than half of the mice treated with the combination therapy (Fig. 6e). Consistent with these findings, combination treatment significantly improved survival rates compared to monotherapy (Fig. 6f).

To evaluate the potential adverse effects on the normal hematopoietic system, Fgd5tm3 mice [33] were crossed with Ai14 mice and subsequently subjected to combined CDK4/6i and ATRA treatment, as outlined in Fig. 6g. Two days after the tamoxifen injection, partial but selective tdTomato expression was observed in lineage^-^c-kit^+^ZsGreen (Fgd5)^+^ hematopoietic stem cells (HSCs) (Fig. 6h). By the end of drug treatment, a reduction in the ZsGreen-derived signal was detected in a minor population of tdTomato-labeled HSCs (Fig. 6h), which is presumed to represent post-HSCs with reduced Fgd5 expression. Under these experimental conditions, the combined drug treatment did not exert any detrimental effects on the hematopoietic system, as evidenced by the unchanged numbers of RBCs (Fig. 6i), WBCs (Fig. 6j), platelets (Fig. 6k), and bone marrow (BM) lineage^-^c-kit^+^Fgd5^+^ HSCs (Fig. 6l). Moreover, the stability of HSCs, represented by ZsGreen expression in tdTomato-labeled cells, remained unaffected following treatment (Fig. 6m). These findings suggest that a combination of CDK4/6i and ATRA is tolerable by the normal hematopoietic system.

## Discussion

We found that the conflicting activation of mitogenic ERK signaling under conditions of cell cycle arrest induces cytotoxicity in heterogenous AML cell lines following combined treatment with ATRA and CDK4/6i palbociclib. Blagosklonny recently proposed that sustained activation of growth signaling in non-proliferating cells contributes to geroconversion during cellular senescence, analogous to simultaneously pressing both the brakes and the accelerator [1]. More recently, several studies have demonstrated that mitogenic mTOR signaling plays a key role in promoting cellular overgrowth during G1 cell cycle arrest, thereby triggering geroconversion [34–37]. In these studies, palbociclib monotherapy effectively induced cellular senescence in human breast cancer cell lines, but not in B cell precursor leukemia cell lines [36]. Consistent with these findings, we observed that palbociclib alone was insufficient to induce cellular senescence in human AML cell lines, as evidenced by re-entry into the cell cycle after drug withdrawal. In contrast, ATRA-mediated ERK signaling synergized with palbociclib and caused significant cytotoxic effects in AML cells. This suggests that the basement levels of mitogenic signals in leukemia cells under palbociclib-induced cell cycle arrest may be insufficient to trigger cytotoxicity, in contrast to breast cancer cells.

Mechanistically, we found that cell cycle arrest during interphase facilitates the continuous reassembly of PML-NBs, with an increased number of PML-NBs undergoing multimerization via SUMO association in an ERK signaling-dependent manner. In contrast, Lim et al. reported that ERK2 promotes Pin1-mediated degradation of PML in breast cancer cells [38]. Similarly, arsenic trioxide induces SUMOylation and subsequent degradation of PML by directly binding to it [39], with both cases likely involving proteasomal degradation and reduced PML protein levels. Consistent with this, we observed that arsenic trioxide treatment decreased PML-NB numbers and failed to reproduce the synergistic cytotoxicity in HL60 cells (data not shown). However, our findings that ERK signaling inhibition attenuated the cytotoxic effects of combination treatment, concurrent with the prevention of PML-NB enlargement, and that ERK activation, in contrast, synergized with palbociclib to promote PML-NB enlargement suggest that ERK-driven enlargement of PML-NBs under G1 arrest contributes to cytotoxicity. We therefore speculate that, under G1 arrest conditions, SUMOylated PML may escape cytoplasm translocation and proteasomal degradation, allowing it to accumulate and enlarge within the nucleus.

PML-NBs are membrane-less organelles characterized by a PML protein shell surrounding an inner core [2]. This unique intranuclear architecture serves as a scaffold for epigenetic regulation of gene expression by recruiting various chromatin-associated factors in response to cellular stress [2]. Both PML and several cargo proteins contain SIM motifs that mediate their recruitment to PML-NBs via SUMO-SIM interactions [40]. Kovatcheva et al. recently reported that palbociclib treatment induces cellular senescence in human liposarcoma LS8817 cells through the formation of senescence-associated heterochromatic foci (SAHF), which are mediated by the recruitment of the chromatin-remodeling enzyme ATRX into PML-NBs [41]. Furthermore, Duarte et al. demonstrated that, during senescence induction, the replacement of canonical histones H3.1 and H3.2 with the variant H3.3 precedes SAHF formation [42]. ATRX/DAXX primarily facilitates H3.3 deposition on silenced, methylated alleles, maintaining repressive marks such as H3K9me3 [43]. In line with previous reports, we found that the combination treatment led to the deposition of both H3.3 and H3K9me3 at the transcription start sites of genes encoding RNA polymerase I subunits in AML cells. This epigenetic alteration was accompanied with a marked downregulation in the transcription of these genes, as well as genes involved in translation. Notably, the combination treatment significantly impaired global protein synthesis in a manner dependent on PML and ATRX. Together, these findings suggest that activated PML-NBs suppress protein synthesis primarily through ATRX-dependent epigenetic regulation.

In our preclinical study using MLL-AF9-driven AML-bearing mice, short-term treatment (10 days) with a combination of palbociclib and ATRA, but not with either agent alone, significantly inhibited AML cell growth. Notably, more than half of the mice treated with combination therapy showed complete clearance of leukemia cells from the PB. In contrast, combination therapy under the same conditions had no discernible adverse effects on the normal hematopoietic system, particularly in Fgd5-expressing HSCs. Gazit et al. identified Fgd5 as a reliable marker of HSCs in the BM and developed a reporter system that specifically labels HSCs in mice [33]. Using this system, we demonstrated that neither the number of HSCs nor their Fgd5 expression levels were affected by the combination treatment. Consistent with the well-established knowledge that palbociclib can induce myelosuppression as a side effect [44], our unpublished data revealed that long-term (28 days) palbociclib monotherapy resulted in a transient reduction in WBC and RBC counts. However, these hematological defects were fully resolved after treatment. Importantly, we observed that the same treatment did not lead to the accumulation of p16-expressing senescent cells in the BM, as assessed in p16-CreERT2-tdTomato mice [45] (manuscript in preparation). Taken together, these findings suggest that the combination of palbociclib and ATRA is not only highly effective, but also a safe therapeutic strategy for AML.

In this study, we identified a novel cell biological phenomenon involving the activation of conflicting cell cycle signals, characterized by the enlargement of PML-NBs induced by mitogenic ERK signaling under cell cycle arrest. A hallmark of neoplasms is the constitutive activation of mitogenic signaling, which is typically elevated compared to that in normal cells, suggesting a preferential targeting of neoplastic cells. Further elucidation of this mechanism may provide a foundation for developing a novel therapeutic strategy with broad applicability across various types of neoplasms.

## Materials and methods

### Mice

Specific pathogen-free 6–8-week-old male BALB/c mice were purchased from Charles River Japan (Kanagawa, Japan). Fgd5tm3(cre/ERT2) mice and Ai14 mice of the C57BL/6 J strain were obtained from Jackson Laboratories (Bar Harbor, ME, USA). All mice were kept under specific pathogen-free conditions, housed (five per cage) in a temperature (21–25 °C), and maintained on a 12 h light/dark cycle (lights on at 08:00 to 20:00). The mice had ad libitum access to standard food and water. All experiments were performed in accordance with relevant guidelines and regulations and complied with the ARRIVE (Animal Research: Reporting of In Vivo Experiments) guidelines. Mouse experiments were not randomized. The investigators were not blinded when separating mice into each experimental group.

### Antibodies (Abs)

The following rat, mouse, or rabbit anti-human monoclonal (m)Abs: anti-CD11b (M1/70; 20-0112, TONBO Biosciences, San Diego, CA), anti-CD38 (HB7; 50–0388, TONBO Biosciences), anti-DAXX (25C12; 4533, Cell Signaling, Danvers, MA), anti-HIRA (EPR7416; ab129169, abcam Cambridge, UK), anti-histone H3.1/2 (1D4F2; CEC-006, Cosmo Bio, Tokyo, Japan), anti-histone H3.3 (4H2D7; CEC-008, Cosmo Bio), anti-PML (C7; ab96051, abcam), anti-pERK1/2 (197G2; 33370, Cell Signaling), and anti-SUMO-2/3 (18H8; 4971, Cell Signaling) were used. Rabbit polyclonal anti-human ATRX (ab97508, Abcam) and PML (21041-1-AP, Proteintech, Rosemont, IL) Abs were also used. APC-conjugated rabbit control mAb (DA1E; 12445) and mouse/rabbit control IgGs were purchased from Cell Signaling and Thermo Fisher Scientific (Waltham, MA), respectively.

### Culture of cell lines, drug treatment, and cell reproliferation assay

Human AML cell lines, MOLM-13 and MOLM-14, were obtained from JCRB Cell Bank (Osaka, Japan). HL60, MV4-11, and NB4 cell lines were purchased from ATCC (Manassas, VA). The experiments utilized frozen stocks of cell lines prepared within a few passages after acquisition. The cell lines were authenticated by their respective cell banks and further authentication was not conducted. A mycoplasma test was not conducted upon receipt from the cell bank. All cell lines were cultured in RPMI-1640 medium supplemented with 10% FBS. The cells were treated with 1 μM ATRA (Sigma-Aldrich, Burlington, MA) alone or in combination with 1 μM of the CDK4/6 inhibitor (CDK4/6i) palbociclib (Selleckchem, Houston, TX). In the reproliferation assay, cells were washed twice with fresh culture medium for the indicated time periods of drug treatment. The cells were then plated in 96-well plates at a density of 10^4^ viable cells per well and their number and viability were determined using trypan blue staining method. Cell proliferation was determined using the Cell Counting Kit-8 (DOJINDO, Kumamoto, Japan) at the indicated time points until confluency was reached.

### Establishment of knock-down and genome-edited cell lines

PML and ATRX knockdown cells were established from HL60 cells by infection with the MISSION lentivirus carrying pLKO puro with shRNA constructs (PML-1: TRCN0000003869, PML-2: TRCN0000314603, ATRX-1: TRCN0000013590, ATRX-2: TRCN0000013588) purchased from Sigma-Aldrich. As a control, HL60 cell line was infected with MISSION lentivirus carrying pLKO puro with non-mammalian shRNA control constructs (SHC002V). For genome editing in the MOLM-13 cell line, the cells were infected with lentiviral particles carrying the U6-gRNA:ef1a-puro-2A-Cas9-2A-tGFP construct (gRNA sequence for PML: GTTCACGCGCAGATGCACG, non-targeting gRNA for scramble: CGCGATAGCGCGAATATATT) purchased from Sigma-Aldrich. Stably transfected cells were selected in culture medium supplemented with 1 μg/ml puromycin for more than 2 weeks.

### Establishment of cell line with doxycycline-inducible activation of ERK signaling

HL60 cells were infected with a lentivirus carrying pCWXPGR-pTF-ERK:MEK, which was a gift from Patrick Salmon (Addgene plasmid # 114291). Seven days post-infection, GFP-expressing cells were isolated using a FACSAria III (BD Biosciences, San Jose, CA).

### Generation of mouse AML model

As described in detail in our previous study [46], total bone marrow (BM) cells were harvested from AML-developing mice transplanted with human MLL-AF9-transduced mouse hematopoietic stem or progenitor cells, and stored in liquid nitrogen as a frozen stock of primary AML cells. To prepare the mouse AML model, 2 × 10^5^ colony forming cells, briefly cultured in semi-solid methylcellulose-based medium (Methocult GF M3534, STEMCELL Technologies, Vancouver, Canada), were intravenously transplanted into sub-lethally irradiated secondary recipient mice along with 1 × 10⁶ normal BM cells.

### In vivo drug administration

ATRA (Sigma-Aldrich) and palbociclib-HCL (Selleckchem) were dissolved in DMSO and distilled water to make a 2.5 and 20 mg/ml stock solutions, respectively. Mice bearing AML were intraperitoneally injected with ATRA (5 mg/kg, diluted in PBS containing 5% polyethylene glycol 400 (Sigma-Aldrich) and 5% Tween 80 (Sigma-Aldrich)) and/or palbociclib-HCL by oral gavage (100 mg/kg, diluted in water).

### NBT reduction assay

One NBT tablet (Sigma-Aldrich) was dissolved in 10 ml culture medium and used as the working solution. Cells were incubated with 10 µg/ml PMA in the NBT working solution for 20 min at 37 °C. The cytospin-prepared slides were fixed with methanol and counterstained with 0.15% safranin O for 30 s.

### RNA extraction and RNA-Seq analysis

Total RNA was extracted from cells using the RNeasy Plus Mini Kit (QIAGEN, Limburg, Netherlands). RNA quality was assessed using a NanoDrop (Thermo Fisher Scientific) and a Qubit 3.0 Fluorometer (Thermo Fisher Scientific). Qualified RNA was subjected to the RNA-Seq library preparation using the NEBNext Ultra II Directional RNA Library Prep Kit for Illumina (New England Biolabs, Ipswich, MA). A NovaSeq 6000 (Illumina, San Diego, CA) was used for sequencing. Raw data were processed using the supercomputing resources provided by the Human Genome Center, Institute of Medical Science, University of Tokyo. RNA-Seq reads for each sample were aligned to the human genome (hg38) using STAR aligner (v2.7.11a) [47]. Aligned reads were subsequently assembled into transcripts guided by reference annotation (NCBI Refseq). RSEM (v1.3.3) was used to obtain the raw gene counts from the read alignments [48]. Differential expression analysis was performed using DESeq2 (v1.36.0) [49].

### Immunofluorescent staining and PLA

The cells were immortalized on a slide using a Cytospin 4 (Thermo Fisher Scientific) and subjected to immunofluorescent (IF) staining for PML-NBs, as well as a PLA using the Duolink In Situ Orange Starter Kit (Merck, Rahway, NJ), according to the manufacturer’s instructions. Specificity of PLA was validated using control IgGs (Supplementary Fig. 13). For IF and PLA, the cells were fixed with 100% methanol for 5 min and 4% paraformaldehyde for 10 min, respectively, and then permeabilized with 0.1% Triton X-100 for 5 min. Images were obtained using a BZ-X710 microscope, and the number of PML-NBs per nucleus, as well as the total signal intensity of fluorescent-positive dots—calculated based on area and brightness—were quantified using Keyence Analysis Software.

### Flow cytometry

Intracellular PML and ATRX were stained with each protein-specific mAb using a Foxp3/Transcription Factor Buffer Set (eBioscience, San Diego, CA). Intracellular phosphorylated ERK1/2 was stained with a specific mAb after secondary fixation with 100% cold methanol for 20 min on ice, followed by primary fixation with Foxp3/Transcription Factor Buffer Set. For the analysis of apoptosis, cell cycle, β-galactosidase activity, and protein synthesis, cells were stained using the Annexin V-FITC Apop Kit (Thermo Fisher Scientific), the Cell Cycle Assay Solution Deep Red Kit (DOJINDO), the CellEvent Senescence Green Flow Cytometry Assay Kit (Thermo Fisher Scientific), and the Click-iT HPG Alexa Fluor Protein Synthesis Assay Kit (Thermo Fisher Scientific), respectively. The expression of each molecule was determined using a FACSCanto II (BD Biosciences) and analyzed with FlowJo software (Tree Star, Ashland, OR).

### Protein extraction and western blotting

The cells were lysed with cold RIPA buffer (Nacalai Tesque, Kyoto, Japan) containing ProteoGuard EDTA-Free Protease Inhibitor Cocktail (Takara, Shiga, Japan) and a Phosphatase Inhibitor Cocktail (Cell Signaling). For the detection of multimerized PML protein, the cell lysate was centrifuged at 9000 g for 5 min at 4 °C after incubation on ice for 10 min. The pellet was then resuspended with PBS containing 250 U/ml benzonase nuclease (Sigma-Aldrich) and incubated for 2 h at 25 °C. Protein concentration was measured using the Qubit Protein Assay Kit (Thermo Fisher Scientific), 1 mg/ml protein samples were loaded into a 66-440 kDa Separation Module (Bio-Techne, Minneapolis, MN), and PML protein was detected using rabbit polyclonal anti-human PML Abs (dilution 1:50). To detect p53 protein phosphorylation and acetylation, the cell lysate was sonicated on ice for 1 min and then incubation on ice for 30 min. It was then centrifuged at 9000 × g for 5 min at 4 °C, and protein concentration was determined using the Pierce Dilution-Free Rapid Gold BCA Protein Assay Kit (Thermo Fisher Scientific). Protein samples at a concentration of 1 mg/ml were loaded into a 12–230 kDa Separation Module (Bio-Techne), and each modification of the p53 protein was detected using a p53 Antibody Sampler Kit (Cell Signaling). Simple western blot analysis was performed using Protein Simple Abby platform (Bio-Techne).

### ChIP-Seq analysis

DNA sample preparation for ChIP-Seq analysis, including crosslinking between proteins and DNA, chromatin fragmentation, immunoprecipitation, and DNA purification, was conducted using the SimpleChIP Enzymatic Chromatin IP Kit (Cell Signaling). DNA quality was assessed with a NanoDrop (Thermo Fisher Scientific) and a Qubit 3.0 Fluorometer (Thermo Fisher Scientific). Qualified DNA was subjected to the ChIP-Seq library preparation using the NEBNext Ultra II DNA Library Prep Kit for Illumina (New England Biolabs). NovaSeq 6000 (Illumina) was used for sequencing. Raw data were processed using supercomputing resources provided by the Human Genome Center, Institute of Medical Science, University of Tokyo. Quality of ChIP-seq datasets was assessed using the FastQC tool (version 0.12.1). Raw ChIP-seq reads were aligned to the human reference genome (hg38) using bowtie2 (version 2.5.2) [50]. Multi-mapping reads were aligned to the optimal location using the alignment score. (If there are same score multimapping score read, that reads were aligned only location randomly). Duplicate reads were filtered using Picard (version 2.21.4). Peak calling was performed with HOMER (version 4.11.1) software [51] using a p-value cutoff of 0.001. GO analysis was performed GREAT [52] using optioned proximal 5.0 kb upstream1.0 kb distal up to 50.0 kb. Dot plot for GO enrichment analysis were generated using custom R script.

### Statistical analysis

Specific sample size calculation methods were not used. Technically validated data were not excluded. Data were statistically analyzed using GraphPad Prism software (Ver. 6; La Jolla, CA) using the methods indicated in the legend of each figure. Two-sided Student’s *t*-test and one-way ANOVA followed by Dunnett or Tukey–Kramer post-hoc tests were used to compare the data between two and more than two groups, respectively. The log-rank test was used to evaluate the survival curve data. Statistical significance was set at *p* < 0.05.

## Supplementary information

Supplementary Figures

## Data Availability

Raw and processed data of RNA-Seq and ChIP-Seq are available at Gene Expression Omnibus (GEO) under accession number GSE276457 and GSE276456, respectively.
