# Supplementary material for: Enlarged PML-nuclear bodies trigger conflicting cell cycle signal-mediated cytotoxicity in leukemia cells
Source: Cell Death Dis. 2025 Aug 2;16(1):586. doi: 10.1038/s41419-025-07911-7 (PMC12318136; doi:10.1038/s41419-025-07911-7)
Supplement: Supplementary file 1 — Supplementary Figures [file 41419_2025_7911_MOESM1_ESM.pdf]

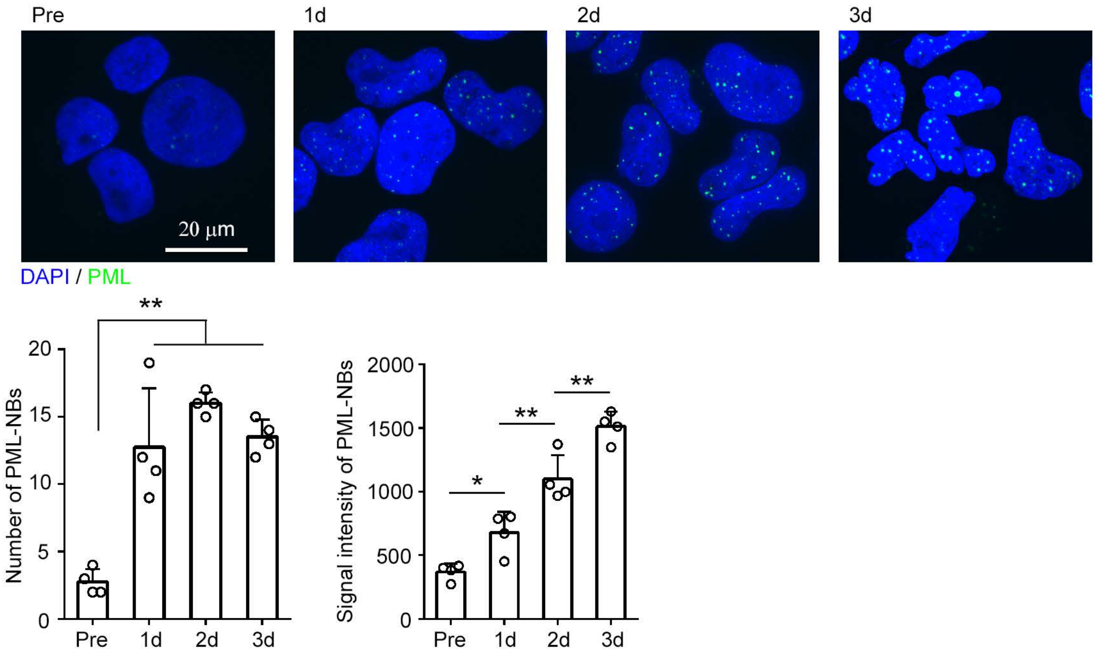

Supplementary Figure 1. ATRA-mediated PML-NB formation in NB4 cells. NB4 cells were treated with 1 µM ATRA, and immunofluorescent staining for PML-NBs was performed at the indicated time points. Representative images are shown, along with the mean + SD of the number of PML-NBs per nucleus and the average of their signal intensity (n = 4). **P < 0.01; *P < 0.05 (Tukey-Kramer's test).

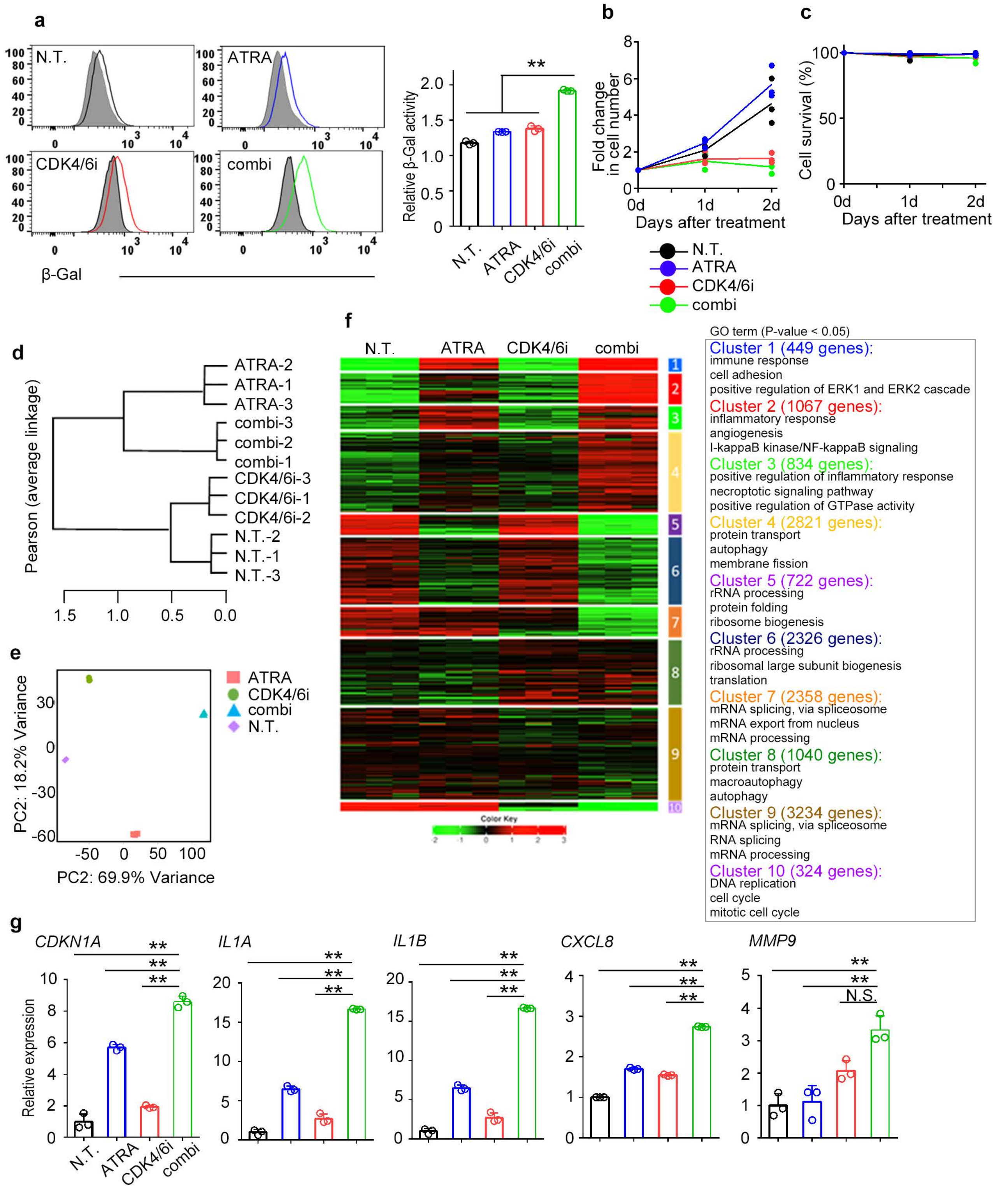

Supplementary Figure 2. Combined treatment with CDK4/6i and ATRA in HL60 cells. HL60 cells were treated with 1 μM CDK4/6i and/or 1 μM ATRA, with non-treated cells serving as controls. (a) β-galactosidase activity was measured 48 h after drug treatment. Representative data and the mean + SD are shown (n = 3). **P < 0.01 (Tukey-Kramer's test). Gray-filled histograms indicate non-staining background. (b) Cell counts were performed at the indicated time points following drug treatment (n = 3). The fold change in cell number was calculated by dividing the number at each time point by the number at day 0. (c) Cell viability was assessed at the indicated time points post-treatment (n = 3), and the percentage of viable cells is presented as cell survival. (d-g) RNA-Seq analysis was conducted 48 h post-treatment. Results from the phylogenetic tree analysis (d), principal component analysis (e), heat map analysis (f), and relative mRNA expression of senescence-related factors (g) are presented (n = 3). The representative GO terms from each cluster in the heat map are highlighted. **P < 0.01; N.S., no significant difference (Tukey-Kramer's test).

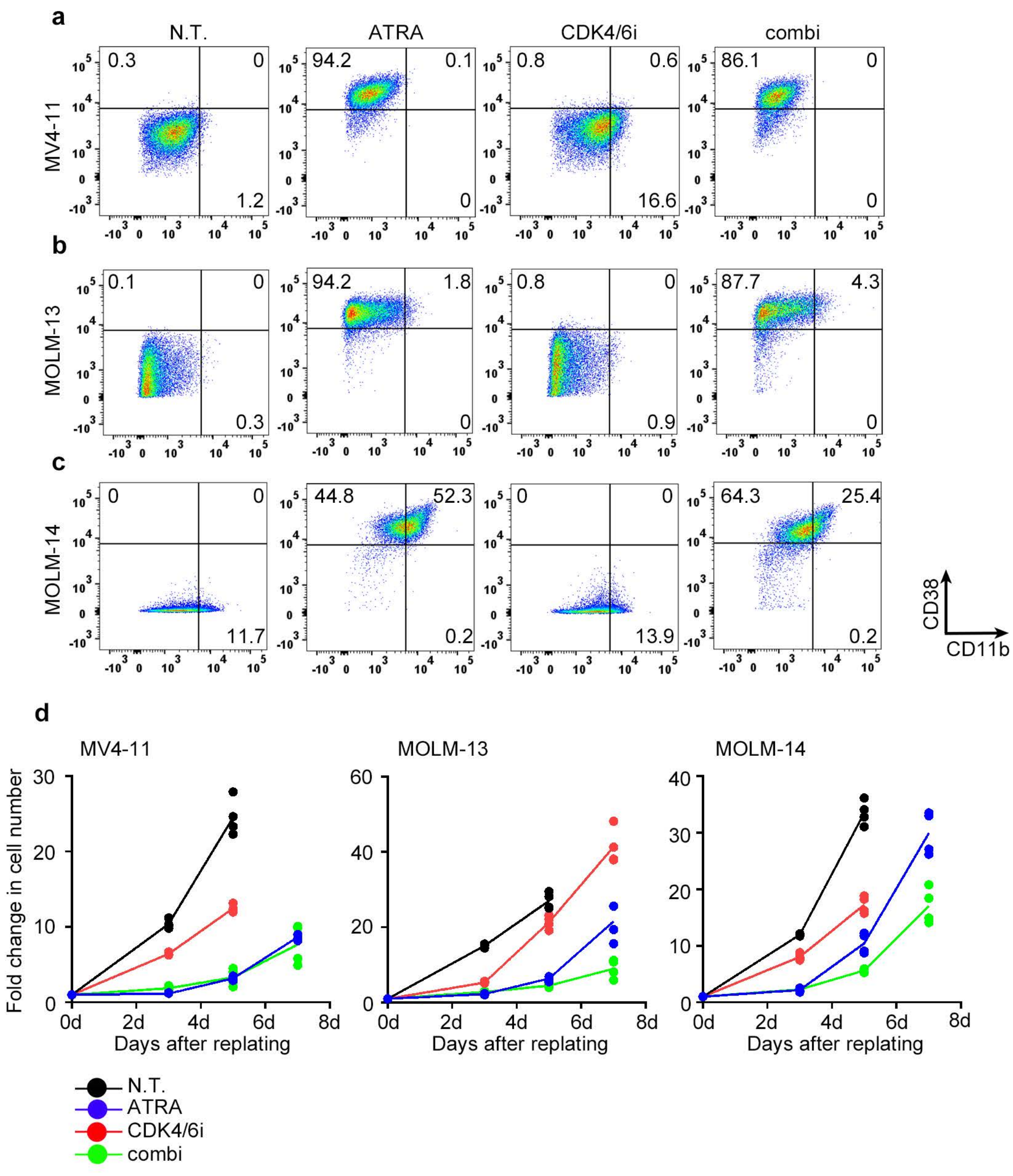

Supplementary Figure 3. Combined treatment with CDK4/6i and ATRA in human acute monoblastic and monocytic leukemia cell lines. MV4-11 cells, MOLM-13 cells, and MOLM-14 cells were treated with 1 μM CDK4/6i and/or 1 μM ATRA, with non-treated cells serving as controls. (a-c) The expression of CD38 and CD11b on cells 48 h after drug treatment. Representative data from three independent experiments are shown. (d) Cell growth was measured in drug-free medium following 48 h of prior drug treatment (n = 4). The fold change in cell number was calculated by dividing the values at each time point by the values at day 0.

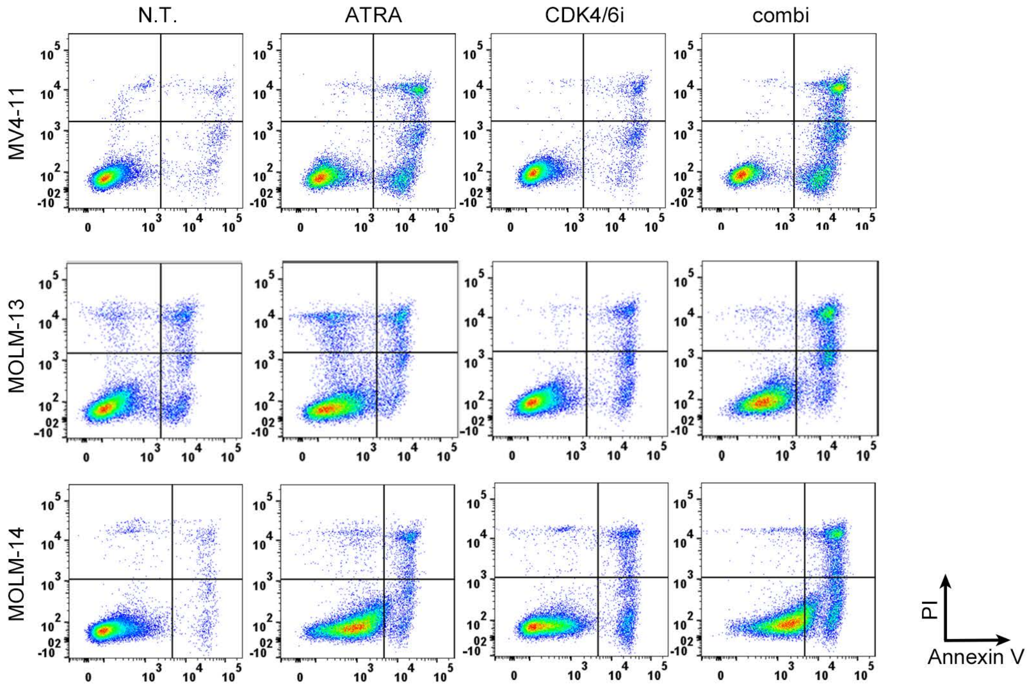

Supplementary Figure 4. Apoptosis induction by combined treatment with CDK4/6i and ATRA in human acute monoblastic and monocytic leukemia cell lines. MV4-11 cells, MOLM-13 cells, and MOLM-14 cells were treated with 1 μM CDK4/6i and/or 1 μM ATRA, with non-treated cells serving as controls. Apoptotic cells were stained using the Annexin V-FITC Apop Kit 48 h after drug treatment. Representative data from three independent experiments are shown.

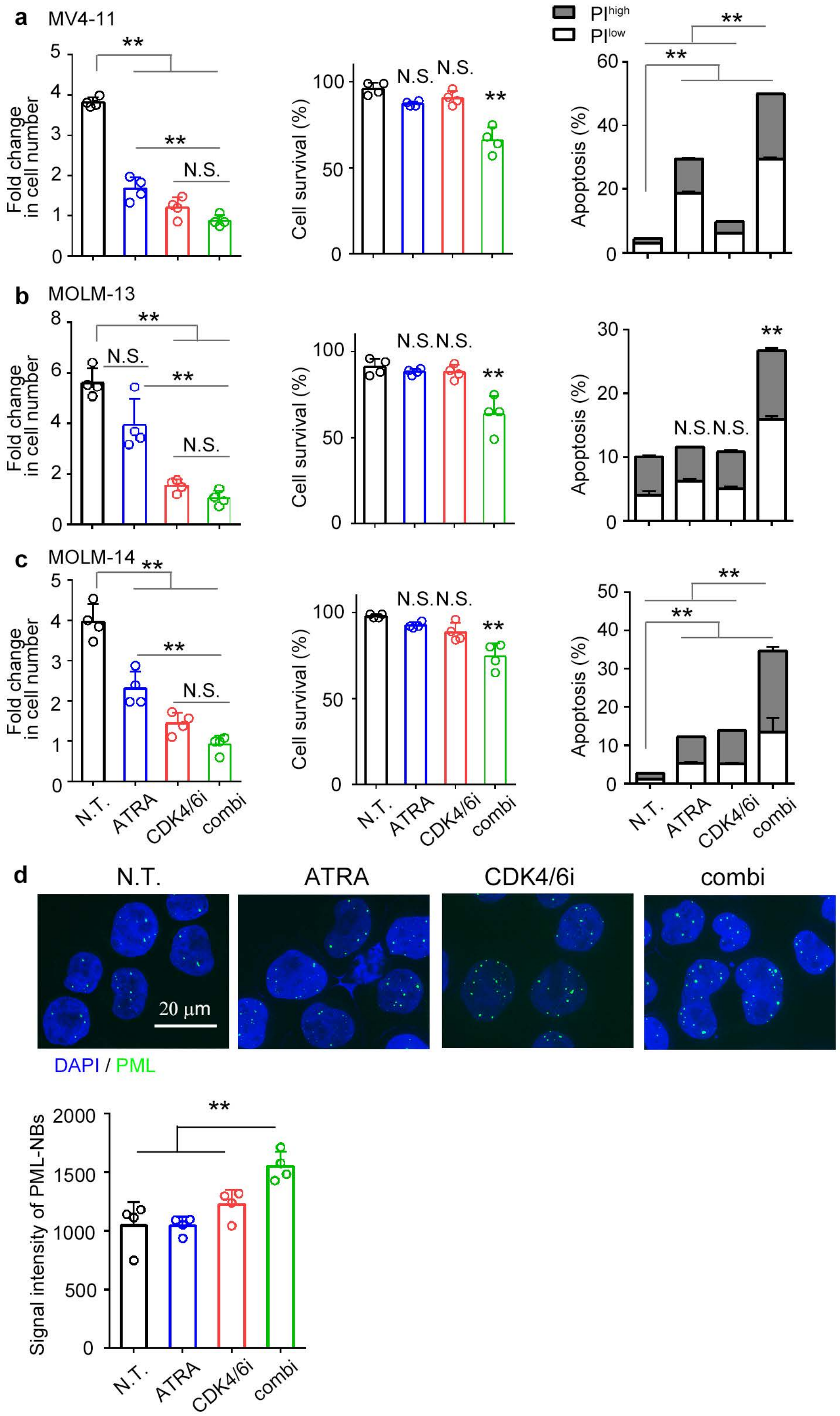

Supplementary Figure 5. Combined CDK4/6i and ATRA treatment induces apoptosis-dependent cell death and promotes PML-NB formation in human acute monoblastic and monocytic leukemia cell lines. MV4-11 cells, MOLM-13 cells, and MOLM-14 cells were treated with 1 μM CDK4/6i and/or 1 μM ATRA, with non-treated cells serving as controls. (a-c) Cell counts, viability, and apoptosis were evaluated 48 h after treatment. The fold change in cell number was calculated by dividing the number at 48 h by the number at day 0. For statistical analysis, the sum of Annexin V+PI+ late apoptotic cells and Annexin V+PI- early apoptotic cells was used. Data are presented as mean + SD from four independent experiments (for cell proliferation and viability) and three independent experiments (for apoptosis). (d) Immunofluorescent staining for PML-NBs was conducted 24 h after treatment. Representative images are shown, along with the mean + SD of the average of signal intensity of PML-NBs (n = 4). **P < 0.01; N.S., no significant difference (Tukey-Kramer's test).

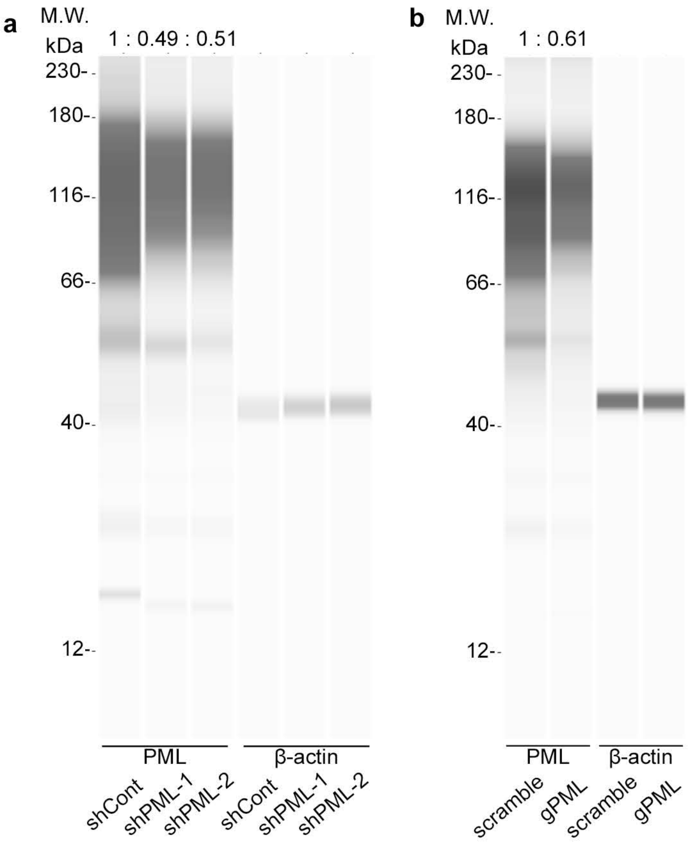

Supplementary Figure 6. Validation of the silencing efficiency of PML gene-specific shRNA and gRNA treatment. Western blot analysis of PML protein expression in (a) HL60 cells transduced with PML-specific shRNA and (b) MOM-13 cells following CRISPR/Cas9-mediated gene editing using PML-targeting gRNA. The relative expression of PML was quantified and normalized to β-actin.

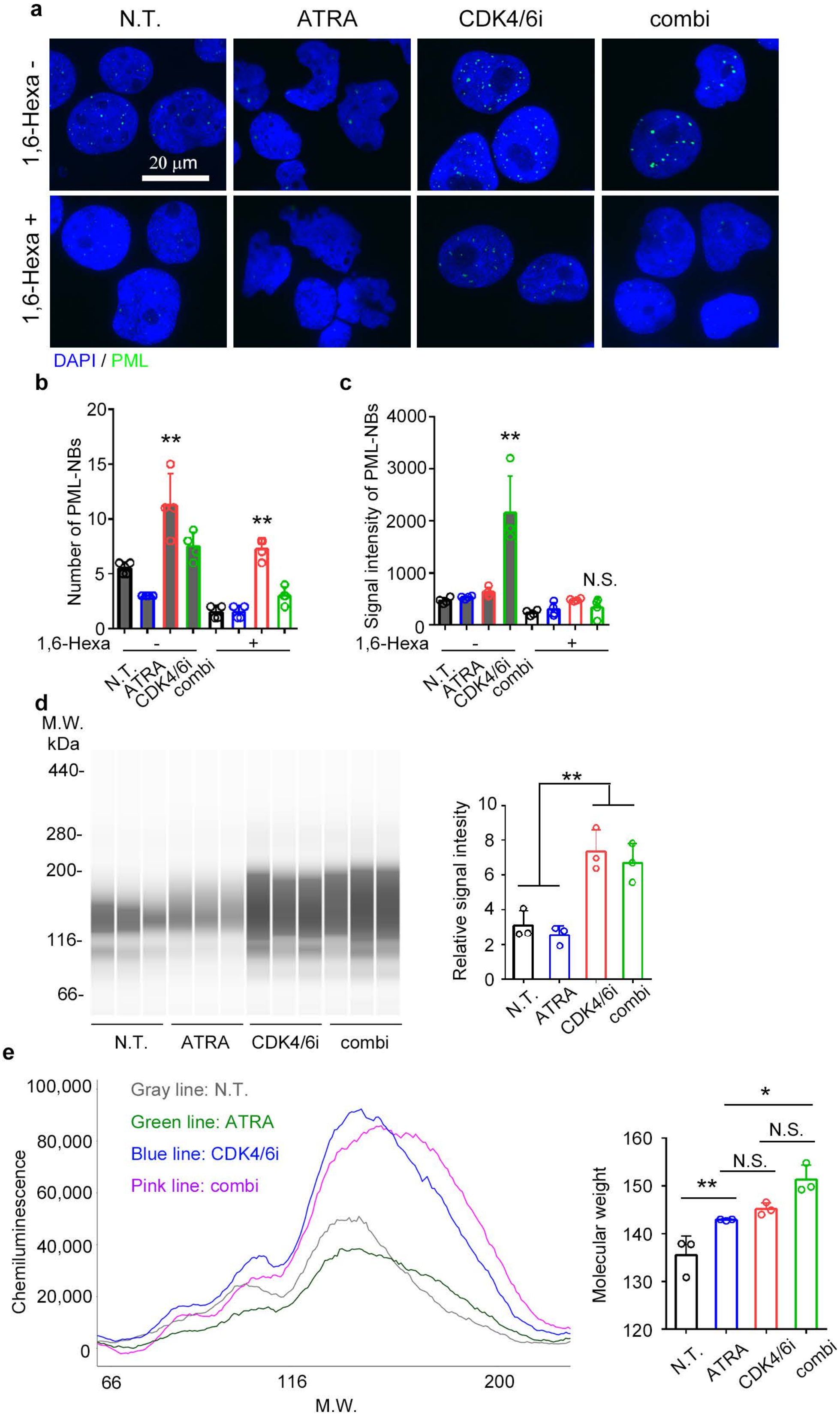

Supplementary Figure 7. Multimerization of PML-NBs induced by combined CDK4/6i and ATRA treatment. HL60 cells were treated with 1 μM CDK4/6i and/or 1 μM ATRA for 48 h, with non-treated cells serving as controls. (a-c) Immunofluorescent staining for PML-NBs was performed following a 10-min incubation in medium with or without 2 % 1,6-hexanediol (WAKO) at room temperature. Representative images are shown, along with the mean + SD for the number of PML-NBs per nucleus and the average of their signal intensity (n = 4). (d, e) Western blot analysis was performed using the RIPA buffer-insoluble protein fraction. Representative blot images are shown, with the mean + SD of signal intensity (d) and the molecular weight distribution (e) of multimerized PML (n = 3). **P < 0.01; *P < 0.05; N.S., no significant difference (Dunnett's test for panels b and c; Tukey-Kramer's test for panels d and e).

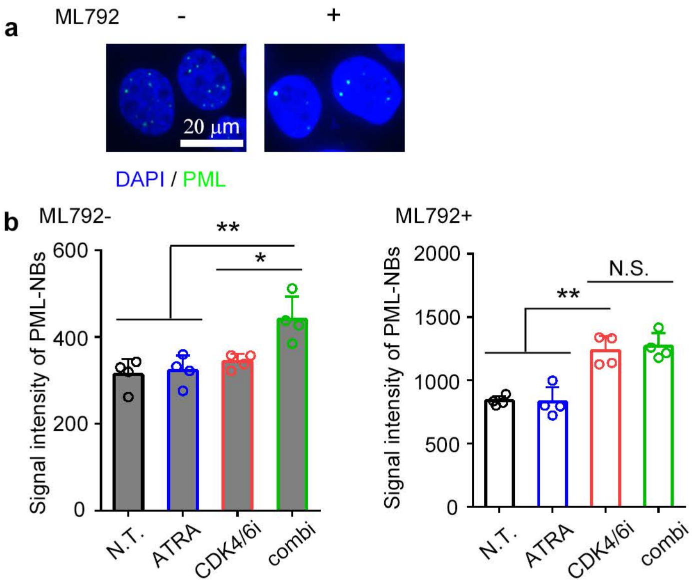

Supplementary Figure 8. ATRA does not further enhance PML-NB signals when combined with CDK4/6i in the presence of ML792. HL60 cells were treated with 1 µM CDK4/6i and/or 1 µM ATRA in the presence or absence of 5µM ML792, with non-treated cells serving as controls. After 24 h of treatment, immunofluorescent staining was performed to visualize PML-NBs. (a) Representative images of control samples treated with or without ML792. (b) Mean + SD for the average of signal intensity of PML-NBs (n = 4). **P < 0.01; *P < 0.05; N.S., no significant difference (Tukey-Kramer's test).

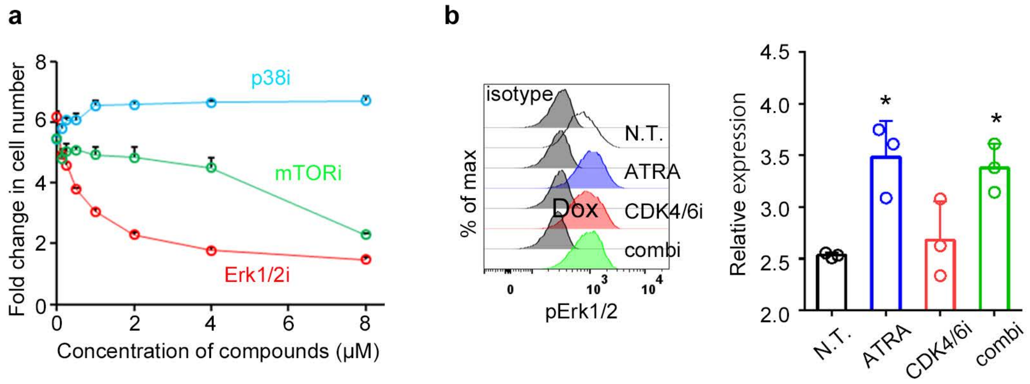

Supplementary Figure 9. ATRA activates ERK signaling in HL60 cells. (a) HL60 cells were treated with serial concentrations of ERK1/2 inhibitor (ravoxertinib), mTOR inhibitor (rapamycin), and p38 MAPK inhibitor (SB203580). Cell numbers were assessed 48 h post-treatment (n = 3). The fold change in cell number was calculated relative to number at day 0. (b) HL60 cells were treated with 1 μM CDK4/6i and/or 1 μM ATRA, with non-treated cells serving as controls. ERK1/2 phosphorylation was assessed 15 h post-treatment. Representative data are shown along with the mean + SD of relative phosphorylated ERK1/2 expression (n = 3), calculated as the ratio of the MFI of anti-pERK1/2 Ab to that of the isotype control. *P < 0.01 (Dunnett's test).

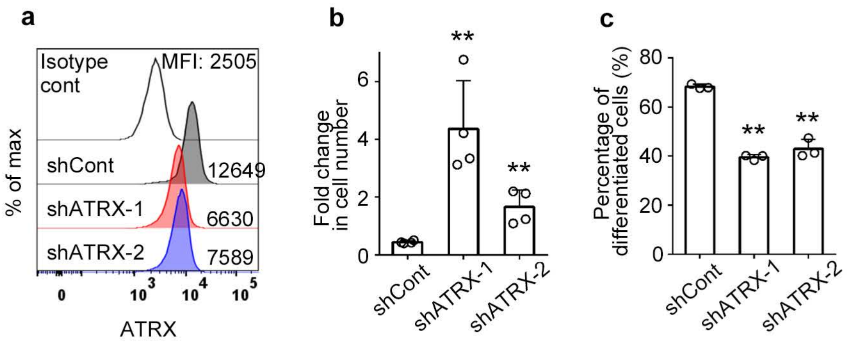

Supplementary Figure 10. ATRX silencing mitigates the cell growth arrest and myeloid differentiation induced by combined CDK4/6i and ATRA treatment. (a) ATRX expression was analyzed in shControl- and shATRX-transduced HL60 cells. Data show the MFI from a representative experiment. (b) Proliferation of shControl- and shATRX-transduced HL60 cells was evaluated in drug-free medium following 48 h of prior CDK4/6i+ATRA treatment. Fold change in cell number was calculated by dividing the values at day 9 by the values at day 0. Data are presented as mean + SD from four independent experiments. (c) The expression of CD38 and CD11b on shControl- and shATRX-transduced HL60 cells were assessed 48 h after CDK4/6i+ATRA treatment. The mean + SD of the percentage of CD11bhighCD38low cells is shown (n = 3). *P < 0.01 (Dunnett's test).

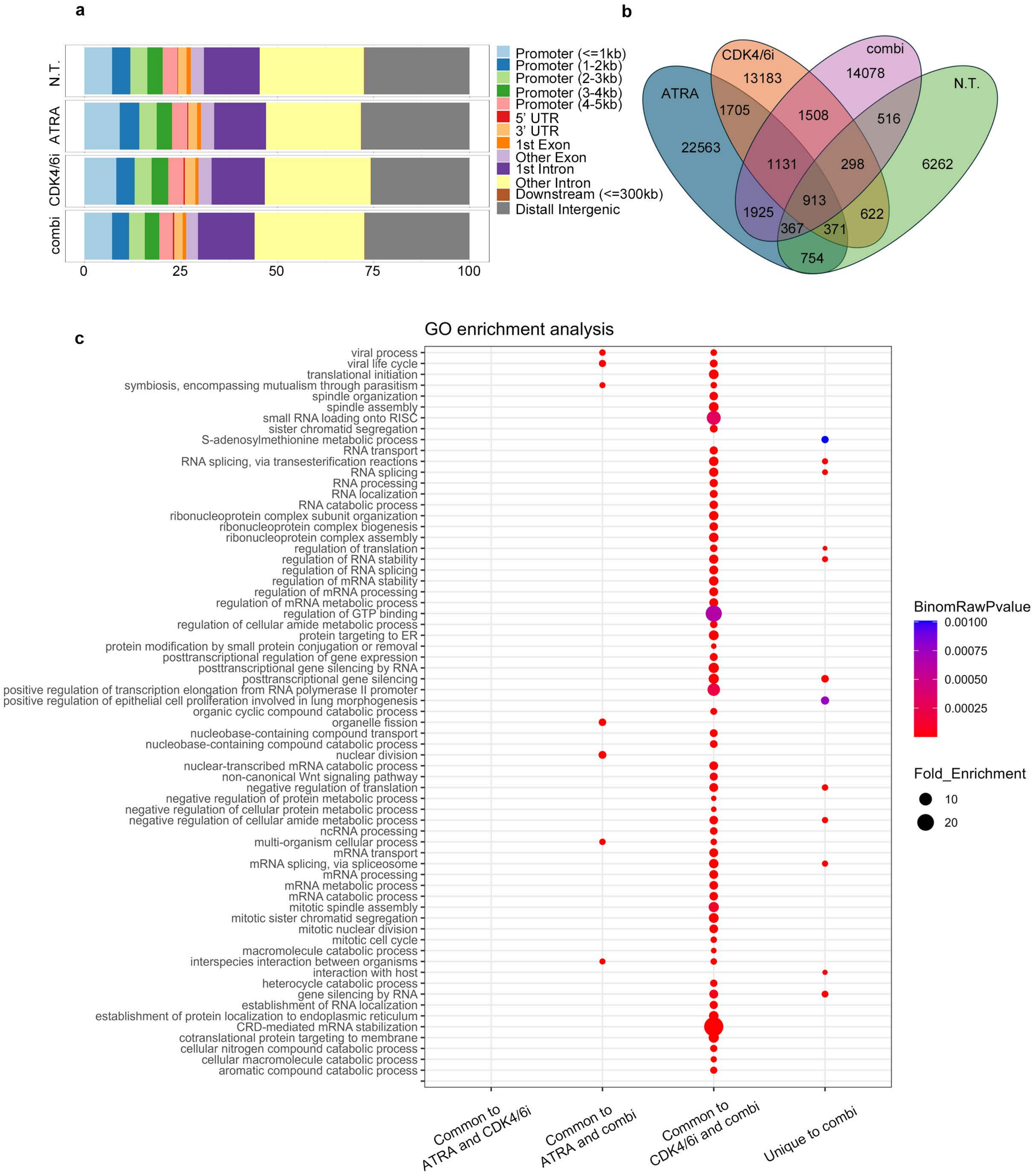

Supplementary Figure 11. Chromatin deposition of histone H3.3 at de novo protein synthesis-related genes is primarily induced by CDK4/6 inhibition and synergistically enhanced by ATRA. HL60 cells were treated with 1 μM CDK4/6i and/or 1 μM ATRA for 48 h, with non-treated cells serving as controls. (a) Distribution of histone H3.3 deposition peaks throughout genetic regions. (b) Venn diagram illustrating the overlap in histone H3.3 deposition peaks across the four experimental conditions. (c) GO enrichment analysis was conducted using four data sets of ChIP-Seq peaks, which were observed under following conditions: common to ATRA and CDK4/6i treatment, common to ATRA and combined treatment, common to CDK4/6i and combined treatment in common, and unique to combined treatment.

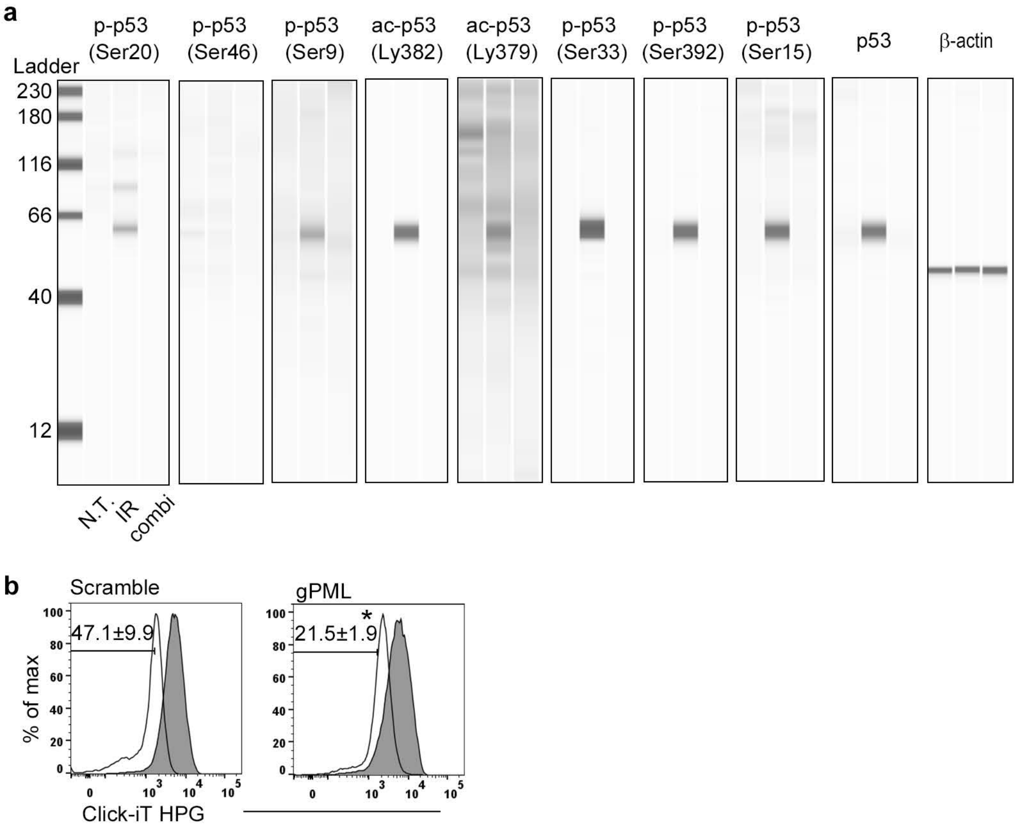

Supplementary Figure 12. p53 signaling is dispensable for the suppression of de novo protein synthesis mediated by combined CDK4/6 inhibition and ATRA treatment in MOLM-13 cells. MOLM-13 cells were treated with 1 μM CDK4/6i and 1 μM ATRA for 24 h, with non-treated cells serving as controls. (a) Western blot analysis was performed using the p53 Antibody Sampler Kit. As a positive control for p53 activation, protein samples were extracted from MOLM-13 cells 2 h post-X-irradiation (25 Gy). Representative blot images from three independent experiments are shown. (b) De novo protein synthesis was assessed 24 h after CDK4/6i+ATRA treatment in non-targeting and PML-specific gRNA-transduced cells using the Click-iT HPG Alexa Fluor Protein Synthesis Assay. Representative data and the mean ± SD of the percentage of cells with impaired protein synthesis are shown (n = 3). *$P < 0.05$ (two-sided Student's t-test).

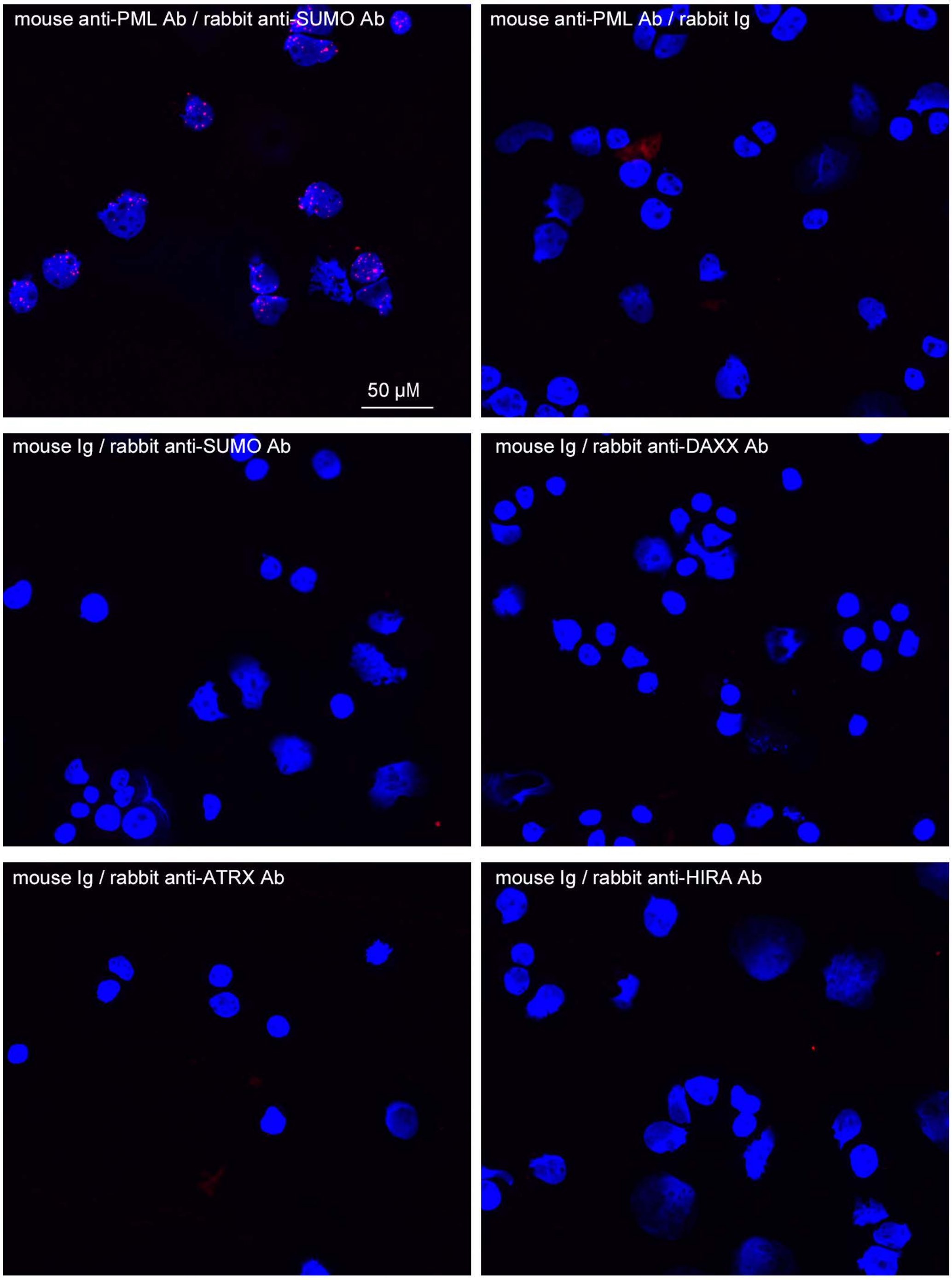

Supplementary Figure 13. Validation of specificity in PLA. HL60 cells immortalized on a slide were subjected to PLA using the indicated protein-specific Ab and control IgGs to validate specificity. Images were captured from three randomly selected fields, and representative images are shown.
